# Immunocompromised patients with acute respiratory distress syndrome: secondary analysis of the LUNG SAFE database

**DOI:** 10.1186/s13054-018-2079-9

**Published:** 2018-06-12

**Authors:** Andrea Cortegiani, Fabiana Madotto, Cesare Gregoretti, Giacomo Bellani, John G. Laffey, Tai Pham, Frank Van Haren, Antonino Giarratano, Massimo Antonelli, Antonio Pesenti, Giacomo Grasselli, LUNG SAFE Investigators and the ESICM Trials Group, Guy M. Francois, Francesca Rabboni, Fabiana Madotto, Sara Conti, John G. Laffey, Giacomo Bellani, Tài Pham, Eddy Fan, Antonio Pesenti, Laurent Brochard, Andres Esteban, Luciano Gattinoni, Frank van Haren, Anders Larsson, Daniel F. McAuley, Marco Ranieri, Gordon Rubenfeld, B. Taylor Thompson, Hermann Wrigge, Arthur S. Slutsky, Fernando Rios, T. Sottiaux, Pieter Depuydt, Fredy S. Lora, Luciano Cesar Azevedo, Guillermo Bugedo, Haibo Qiu, Marcos Gonzalez, Juan I. Silesky Jimenez, Vladimir Cerny, Jonas Nielsen, Manuel Jibaja, Dimitrios Matamis, Jorge Luis Ranero, Pravin Amin, Seyed Mohammadreza Hashemian, Kevin P. Clarkson, Kiyoyasu Kurahashi, Asisclo J. Villagomez, Amine Ali Zeggwagh, Leo M. Heunks, Jon Henrik Laake, Jose Emmanuel Palo, Antero do Vale Fernandes, Dorel Sandesc, Yaseen M. Arabi, Vesna Bumbasirevic, Nicolás Nin, Jose A. Lorente, Lise Piquilloud, Fekri Abroug, Lia McNamee, Javier Hurtado, Ed Bajwa, Gabriel Démpair, Hektor Sula, Lordian Nunci, Alma Cani, Alan Zazu, Christian Dellera, Carolina S. Insaurralde, Risso V. Alejandro, Julio Daldin, Mauricio Vinzio, Ruben O. Fernandez, Luis P. Cardonnet, Lisandro R. Bettini, Mariano Carboni Bisso, Emilio M. Osman, Mariano G. Setten, Pablo Lovazzano, Javier Alvarez, Veronica Villar, Norberto C. Pozo, Nicolas Grubissich, Gustavo A. Plotnikow, Daniela N. Vasquez, Santiago Ilutovich, Norberto Tiribelli, Ariel Chena, Carlos A. Pellegrini, María G. Saenz, Elisa Estenssoro, Matias Brizuela, Hernan Gianinetto, Pablo E. Gomez, Valeria I. Cerrato, Marco G. Bezzi, Silvina A. Borello, Flavia A. Loiacono, Adriana M. Fernandez, Serena Knowles, Claire Reynolds, Deborah M. Inskip, Jennene J. Miller, Jing Kong, Christina Whitehead, Shailesh Bihari, Aylin Seven, Amanda Krstevski, Helen J. Rodgers, Rebecca T. Millar, Toni E. Mckenna, Irene M. Bailey, Gabrielle C. Hanlon, Anders Aneman, Joan M. Lynch, Raman Azad, John Neal, Paul W. Woods, Brigit L. Roberts, Mark R. Kol, Helen S. Wong, Katharina C. Riss, Thomas Staudinger, Xavier Wittebole, Caroline Berghe, Pierre A. Bulpa, Alain M. Dive, Rik Verstraete, Herve Lebbinck, Joris Vermassen, Meersseman Philippe, Helga Ceunen, Jonas I. Rosa, Daniel O. Beraldo, Claudio Piras, Adenilton M. Rampinelli, Antonio P. Nassar, Sergio Mataloun, Marcelo Moock, Marlus M. Thompson, Claudio H. Gonçalves, Ana Carolina P. Antônio, Aline Ascoli, Rodrigo S. Biondi, Danielle C. Fontenele, Danielle Nobrega, Vanessa M. Sales, Suresh Shindhe, Dk Maizatul Aiman, B. Pg Hj Ismail, Francois Beloncle, Kyle G. Davies, Rob Cirone, Venika Manoharan, Mehvish Ismail, Ewan C. Goligher, Mandeep Jassal, Erin Nishikawa, Areej Javeed, Gerard Curley, Nuttapol Rittayamai, Matteo Parotto, Niall D. Ferguson, Sangeeta Mehta, Jenny Knoll, Antoine Pronovost, Sergio Canestrini, Alejandro R. Bruhn, Patricio H. Garcia, Felipe A. Aliaga, Pamela A. Farías, Jacob S. Yumha, Claudia A. Ortiz, Javier E. Salas, Alejandro A. Saez, Luis D. Vega, Eduardo F. Labarca, Felipe T. Martinez, Nicolás G. Carreño, Pilar Lora, Haitao Liu, Ling Liu, Rui Tang, Xiaoming Luo, Youzhong An, Huiying Zhao, Gao Yan, Zhai Zhe, Zheng L. Ye, Wei Wang, Wenwen Li, Qingdong Li, Ruiqiang Zheng, Wenkui Yu, Juanhong Shen, Xinyu Li, Tao Yu, Weihua Lu, Ya Q. Wu, Xiao B. Huang, Yuanhua Lu, Hui Han, Fan Zhang, Renhua Sun, Hua X. Wang, Shu H. Qin, Bao H. Zhu, Jun Zhao, Jian Liu, Bin Li, Jing L. Liu, Fa C. Zhou, Qiong J. Li, Xing Y. Zhang, Zhou Li-Xin, Qiang Xin-Hua, Liangyan Jiang, Yuan N. Gao, Xian Y. Zhao, Yuan Y. Li, Xiao L. Li, Chunting Wang, Qingchun Yao, Rongguo Yu, Kai Chen, Huanzhang Shao, Bingyu Qin, Qing Q. Huang, Wei H. Zhu, Ai Y. Hang, Ma X. Hua, Yimin Li, Yonghao Xu, Yu D. Di, Long L. Ling, Tie H. Qin, Shou H. Wang, Junping Qin, Yi Han, Suming Zhou, Monica P. Vargas, Manuel A. González Rojas, Jaime E. Solis-Quesada, Christian M. Ramirez-Alfaro, Jan Máca, Peter Sklienka, Jakob Gjedsted, Aage Christiansen, Boris G. Villamagua, Miguel Llano, Philippe Burtin, Gautier Buzancais, Pascal Beuret, Nicolas Pelletier, Satar Mortaza, Alain Mercat, Jonathan Chelly, Sébastien Jochmans, Nicolas Terzi, Cédric Daubin, Guillaume Carteaux, Nicolas de Prost, Jean-Daniel Chiche, Fabrice Daviaud, Muriel Fartoukh, Guillaume Barberet, Jerome Biehler, Jean Dellamonica, Denis Doyen, Jean-Michel Arnal, Anais Briquet, Sami Hraiech, Laurent Papazian, Arnaud Follin, Damien Roux, Jonathan Messika, Evangelos Kalaitzis, Laurence Dangers, Alain Combes, Siu-Ming Au, Gaetan Béduneau, Dorothée Carpentier, Elie H. Zogheib, Herve Dupont, Sylvie Ricome, Francesco L. Santoli, Sebastien L. Besset, Philippe Michel, Bruno Gelée, Pierre-Eric Danin, Bernard Goubaux, Philippe J. Crova, Nga T. Phan, Frantz Berkelmans, Julio C. Badie, Romain Tapponnier, Josette Gally, Samy Khebbeb, Jean-Etienne Herbrecht, Francis Schneider, Pierre-Louis M. Declercq, Jean-Philippe Rigaud, Jacques Duranteau, Anatole Harrois, Russell Chabanne, Julien Marin, Charlene Bigot, Sandrine Thibault, Mohammed Ghazi, Messabi Boukhazna, Salem Ould Zein, Jack R. Richecoeur, Daniele M. Combaux, Fabien Grelon, Charlene Le Moal, Elise P. Sauvadet, Adrien Robine, Virginie Lemiale, Danielle Reuter, Martin Dres, Alexandre Demoule, Dany Goldgran-Toledano, Loredana Baboi, Claude Guérin, Ralph Lohner, Jens Kraßler, Susanne Schäfer, Kai D. Zacharowski, Patrick Meybohm, Andreas W. Reske, Philipp Simon, Hans-Bernd F. Hopf, Michael Schuetz, Thomas Baltus, Metaxia N. Papanikolaou, Theonymfi G. Papavasilopoulou, Giannis A. Zacharas, Vasilis Ourailogloy, Eleni K. Mouloudi, Eleni V. Massa, Eva O. Nagy, Electra E. Stamou, Ellada V. Kiourtzieva, Marina A. Oikonomou, Luis E. Avila, Cesar A. Cortez, Johanna E. Citalán, Sameer A. Jog, Safal D. Sable, Bhagyesh Shah, Mohan Gurjar, Arvind K. Baronia, Mohammedfaruk Memon, Radhakrishnan Muthuchellappan, Venkatapura J. Ramesh, Anitha Shenoy, Ramesh Unnikrishnan, Subhal B. Dixit, Rachana V. Rhayakar, Nagarajan Ramakrishnan, Vallish K. Bhardwaj, Heera L. Mahto, Sudha V. Sagar, Vijayanand Palaniswamy, Deeban Ganesan, Hamidreza Jamaati, Farshad Heidari, Edel A. Meaney, Alistair Nichol, Karl M. Knapman, Donall O’Croinin, Eimhin S. Dunne, Dorothy M. Breen, Rola F. Jaafar, Rory Dwyer, Fahd Amir, Olaitan O. Ajetunmobi, Aogan C. O’Muircheartaigh, Colin S. Black, Nuala Treanor, Daniel V. Collins, Wahid Altaf, Gianluca Zani, Maurizio Fusari, Savino Spadaro, Carlo A. Volta, Romano Graziani, Barbara Brunettini, Salvatore Palmese, Paolo Formenti, Michele Umbrello, Andrea Lombardo, Elisabetta Pecci, Marco Botteri, Monica Savioli, Alessandro Protti, Alessia Mattei, Lorenzo Schiavoni, Andrea Tinnirello, Manuel Todeschini, Antonino Giarratano, Andrea Cortegiani, Sara Sher, Anna Rossi, Massimo M. Antonelli, Luca M. Montini, Paolo Casalena, Sergio Scafetti, Giovanna Panarello, Giovanna Occhipinti, Nicolò Patroniti, Matteo Pozzi, Roberto R. Biscione, Michela M. Poli, Ferdinando Raimondi, Daniela Albiero, Giulia Crapelli, Eduardo Beck, Vincenzo Pota, Vincenzo Schiavone, Alexandre Molin, Fabio Tarantino, Giacomo Monti, Elena Frati, Lucia Mirabella, Gilda Cinnella, Tommaso Fossali, Riccardo Colombo, Pierpaolo Terragni, Ilaria Pattarino, Francesco Mojoli, Antonio Braschi, Erika E. Borotto, Andrea N. Cracchiolo, Daniela M. Palma, Francesco Raponi, Giuseppe Foti, Ettore R. Vascotto, Andrea Coppadoro, Luca Brazzi, Leda Floris, Giorgio A. Iotti, Aaron Venti, Osamu Yamaguchi, Shunsuke Takagi, Hiroki N. Maeyama, Eizo Watanabe, Yoshihiro Yamaji, Kazuyoshi Shimizu, Kyoko Shiozaki, Satoru Futami, Sekine Ryosuke, Koji Saito, Yoshinobu Kameyama, Keiko Ueno, Masayo Izawa, Nao Okuda, Hiroyuki Suzuki, Tomofumi Harasawa, Michitaka Nasu, Tadaaki Takada, Fumihito Ito, Shin Nunomiya, Kansuke Koyama, Toshikazu Abe, Kohkichi Andoh, Kohei Kusumoto, Akira Hirata, Akihiro Takaba, Hiroyasu Kimura, Shuhei Matsumoto, Ushio Higashijima, Hiroyuki Honda, Nobumasa Aoki, Hiroshi Imai, Yasuaki Ogino, Ichiko Mizuguchi, Kazuya Ichikado, Kenichi Nitta, Katsunori Mochizuki, Tomoaki Hashida, Hiroyuki Tanaka, Tomoyuki Nakamura, Daisuke Niimi, Takeshi Ueda, Yozo Kashiwa, Akinori Uchiyama, Olegs Sabelnikovs, Peteris Oss, Youssef Haddad, Kong Y. Liew, Silvio A. Ñamendys-Silva, Yves D. Jarquin-Badiola, Luis A. Sanchez-Hurtado, Saira S. Gomez-Flores, Maria C. Marin, Jordana S. Lemus, Jonathan M. Fierro, Mavy Ramirez Cervantes, Francisco Javier Flores Mejia, Dulce Dector, Dulce M. Dector, Daniel R. Gonzalez, Claudia R. Estrella, Jorge R. Sanchez-Medina, Alvaro Ramirez-Gutierrez, Fernando G. George, Janet S. Aguirre, Juan A. Buensuseso, Manuel Poblano, Tarek Dendane, Hicham Balkhi, Mina Elkhayari, Nacer Samkaoui, Hanane Ezzouine, Abdellatif Benslama, Mourad Amor, Wajdi Maazouzi, Nedim Cimic, Oliver Beck, Monique M. Bruns, Jeroen A. Schouten, Myra Rinia, Monique Raaijmakers, Hellen M. Van Wezel, Serge J. Heines, Ulrich Strauch, Marc P. Buise, Fabienne D. Simonis, Marcus J. Schultz, Jennifer C. Goodson, Troy S. Browne, Leanlove Navarra, Anna Hunt, Robyn A. Hutchison, Mathew B. Bailey, Lynette Newby, Colin Mcarthur, Michael Kalkoff, Alex McLeod, Jonathan Casement, Danielle J. Hacking, Finn H. Andersen, Merete S. Dolva, Andreas Barratt-Due, Kim Andre L. Noremark, Eldar Søreide, Brit Å. Sjøbø, Anne B. Guttormsen, Hector H. Leon Yoshido, Ronald Zumaran Aguilar, Fredy A. Montes Oscanoa, Alain U. Alisasis, Joanne B. Robles, Rossini Abbie B. Pasanting-Lim, Beatriz C. Tan, Pawel Andruszkiewicz, Karina Jakubowska, Cristina M. Coxo, António M. Alvarez, Bruno S. Oliveira, Gustavo M. Montanha, Nelson C. Barros, Carlos S. Pereira, António M. Messias, Jorge M. Monteiro, Ana M. Araujo, Nuno T. Catorze, Susan M. Marum, Maria J. Bouw, Rui M. Gomes, Vania A. Brito, Silvia Castro, Joana M. Estilita, Filipa M. Barros, Isabel M. Serra, Aurelia M. Martinho, Dana R. Tomescu, Alexandra Marcu, Ovidiu H. Bedreag, Marius Papurica, Dan E. Corneci, Silvius Ioan Negoita, Evgeny Grigoriev, Alexey I. Gritsan, Andrey A. Gazenkampf, Ghaleb Almekhlafi, Mohamad M. Albarrak, Ghanem M. Mustafa, Khalid A. Maghrabi, Nawal Salahuddin, Tharwat M. Aisa, Ahmed S. Al Jabbary, Edgardo E. Tabhan, Olivia A. Trinidad, Hasan M. Al Dorzi, Stefan Bolon, Oliver Smith, Jordi Mancebo, Hernan Aguirre-Bermeo, Juan C. Lopez-Delgado, Francisco Esteve, Gemma Rialp, Catalina Forteza, Candelaria De Haro, Antonio Artigas, Guillermo M. Albaiceta, Sara De Cima-Iglesias, Leticia Seoane-Quiroga, Alexandra Ceniceros-Barros, Antonio L. Ruiz-Aguilar, Luis M. Claraco-Vega, Juan Alfonso Soler, Maria del Carmen Lorente, Cecilia Hermosa, Federico Gordo, Miryam Prieto-González, Juan B. López-Messa, Manuel P. Perez, Cesar P. Perez, Raquel Montoiro Allue, Ferran Roche-Campo, Marco Ibañez-Santacruz, Susana Temprano, Maria C. Pintado, Raul De Pablo, Pilar Ricart Aroa Gómez, Silvia Rodriguez Ruiz, Silvia Iglesias Moles, Maria Teresa Jurado, Alfons Arizmendi, Enrique A. Piacentini, Nieves Franco, Teresa Honrubia, Meisy Perez Cheng, Elena Perez Losada, Javier Blanco, Luis J. Yuste, Cecilia Carbayo-Gorriz, Francisca G. Cazorla-Barranquero, Javier G. Alonso, Rosa S. Alda, Ángela Algaba, Gonzalo Navarro, Enrique Cereijo, Esther Diaz-Rodriguez, Diego Pastor Marcos, Laura Alvarez Montero, Luis Herrera Para, Roberto Jimenez Sanchez, Miguel Angel Blasco Navalpotro, Ricardo Diaz Abad, Raquel Montiel González, Dácil Parrilla Toribio, Alejandro G. Castro, Maria Jose D. Artiga, Oscar Penuelas, Tomas P. Roser, Moreno F. Olga, Elena Gallego Curto, Rocío Manzano Sánchez, Vallverdu P. Imma, Garcia M. Elisabet, Laura Claverias, Monica Magret, Ana M. Pellicer, Lucia L. Rodriguez, Jesús Sánchez-Ballesteros, Ángela González-Salamanca, Antonio G. Jimenez, Francisco P. Huerta, Juan Carlos J. Sotillo Diaz, Esther Bermejo Lopez, David D. Llinares Moya, Alec A. Tallet Alfonso, Palazon Sanchez Eugenio Luis, Palazon Sanchez Cesar, Sánchez I. Rafael, Corcoles G. Virgilio, Noelia N. Recio, Richard O. Adamsson, Christian C. Rylander, Bernhard Holzgraefe, Lars M. Broman, Joanna Wessbergh, Linnea Persson, Fredrik Schiöler, Hans Kedelv, Anna Oscarsson Tibblin, Henrik Appelberg, Lars Hedlund, Johan Helleberg, Karin E. Eriksson, Rita Glietsch, Niklas Larsson, Ingela Nygren, Silvia L. Nunes, Anna-Karin Morin, Thomas Kander, Anne Adolfsson, Hervé O. Zender, Corinne Leemann-Refondini, Souheil Elatrous, Slaheddine Bouchoucha, Imed Chouchene, Islem Ouanes, Asma Ben Souissi, Salma Kamoun, Oktay Demirkiran, Mustafa Aker, Emre Erbabacan, Ilkay Ceylan, Nermin Kelebek Girgin, Menekse Ozcelik, Necmettin Ünal, Basak Ceyda Meco, Onat O. Akyol, Suleyman S. Derman, Barry Kennedy, Ken Parhar, Latha Srinivasa, Phil Hopkins, Clare Mellis, Vivek Kakar, Dan Hadfield, Andre Vercueil, Kaushik Bhowmick, Sally K. Humphreys, Andrew Ferguson, Raymond Mckee, Ashok S. Raj, Danielle A. Fawkes, Philip Watt, Linda Twohey, Rajeev R. Jha, Matthew Thomas, Alex Morton, Varsha Kadaba, Mark J. Smith, Anil P. Hormis, Santhana G. Kannan, Miriam Namih, Henrik Reschreiter, Julie Camsooksai, Alek Kumar, Szabolcs Rugonfalvi, Christopher Nutt, Orla Oneill, Colette Seasman, Ged Dempsey, Christopher J. Scott, Helen E. Ellis, Stuart McKechnie, Paula J. Hutton, Nora N. Di Tomasso, Michela N. Vitale, Ruth O. Griffin, Michael N. Dean, Julius H. Cranshaw, Emma L. Willett, Nicholas Ioannou, Sarah Gillis, Peter Csabi, Rosaleen Macfadyen, Heidi Dawson, Pieter D. Preez, Alexandra J. Williams, Owen Boyd, Laura Ortiz-Ruiz De Gordoa, Jon Bramall, Sophie Symmonds, Simon K. Chau, Tim Wenham, Tamas Szakmany, Piroska Toth-Tarsoly, Katie H. McCalman, Peter Alexander, Lorraine Stephenson, Thomas Collyer, Rhiannon Chapman, Raphael Cooper, Russell M. Allan, Malcolm Sim, David W. Wrathall, Donald A. Irvine, Kim S. Zantua, John C. Adams, Andrew J. Burtenshaw, Gareth P. Sellors, Ingeborg D. Welters, Karen E. Williams, Robert J. Hessell, Matthew G. Oldroyd, Ceri E. Battle, Suresh Pillai, Istvan Kajtor, Mageswaran Sivashanmugavel, Sinead C. Okane, Adrian Donnelly, Aniko D. Frigyik, Jon P. Careless, Martin M. May, Richard Stewart, T. John Trinder, Samantha J. Hagan, Matt P. Wise, Jade M. Cole, Caroline C. MacFie, Anna T. Dowling, Edgardo Nuñez, Gustavo Pittini, Ruben Rodriguez, María C. Imperio, Cristina Santos, Ana G. França, Alejandro Ebeid, Alberto Deicas, Carolina Serra, Aditya Uppalapati, Ghassan Kamel, Valerie M. Banner-Goodspeed, Jeremy R. Beitler, Satyanarayana Reddy Mukkera, Shreedhar Kulkarni, Jarone Lee, Tomaz Mesar, John O. Shinn III, Dina Gomaa, Christopher Tainter, Dale J. Yeatts, Jessica Warren, Michael J. Lanspa, Russel R. Miller, Colin K. Grissom, Samuel M. Brown, Philippe R. Bauer, Ryan J. Gosselin, Barrett T. Kitch, Jason E. Cohen, Scott H. Beegle, Renaud M. Gueret, Aiman Tulaimat, Shazia Choudry, William Stigler, Hitesh Batra, Nidhi G. Huff, Keith D. Lamb, Trevor W. Oetting, Nicholas M. Mohr, Claine Judy, Shigeki Saito, Fayez M. Kheir, Adam B. Schlichting, Angela Delsing, Daniel . Crouch, Mary Elmasri, Dina Ismail, Kyle R. Dreyer, Thomas C. Blakeman, Rebecca M. Baron, Carolina Quintana Grijalba, Peter C. Hou, Raghu Seethala, Imo Aisiku, Galen Henderson, Gyorgy Frendl, Sen-Kuang Hou, Robert L. Owens, Ashley Schomer, Bojan Jovanovic, Maja Surbatovic, Milic Veljovic

**Affiliations:** 1 Department of Biopathology and Medical Biotechnologies (DIBIMED), Section of Anesthesia, Analgesia, Intensive Care and Emergency, Policlinico Paolo Giaccone, University of Palermo, Via del vespro 129, 90127 Palermo, Italy; 2 Research Center on Public Health, Department of Medicine and Surgery, University of Milano-Bicocca, Monza, Italy; 3 School of Medicine and Surgery, University of Milano-Bicocca, Monza, Italy; 4 Department of Emergency and Intensive Care, San Gerardo Hospital, Monza, Italy; 5 Anesthesia, School of Medicine, National University of Ireland, Galway, Ireland; 6 Interdepartemental Division of Critical Care Medicine, University of Toronto, Toronto, ON Canada; 7 Keenan Research Center for Biomedical Science, Li Ka Shing Knowledge Institute, St. Michael’s Hospital, Toronto, Canada; 8 College of Medicine, Biology and Environment, Australian National University, Canberra, Australia; 9 Intensive Care Unit, Canberra Hospital, Canberra, Australia; 10 Department of Anesthesiology and Intensive Care, Università Cattolica del Sacro Cuore – Fondazione Policlinico Universitario A. Gemelli, Rome, Italy; 11 Department of Anesthesiology, Intensive Care and Emergency, Fondazione IRCCS Ca’ Granda Ospedale Maggiore Policlinico, Milan, Italy; 12 Department of Pathophysiology and Transplantation, University of Milan, Milan, Italy

**Keywords:** Acute respiratory failure, ARDS, Immunocompromised patients, Mechanical ventilation, Noninvasive ventilation

## Abstract

**Background:**

The aim of this study was to describe data on epidemiology, ventilatory management, and outcome of acute respiratory distress syndrome (ARDS) in immunocompromised patients.

**Methods:**

We performed a post hoc analysis on the cohort of immunocompromised patients enrolled in the Large Observational Study to Understand the Global Impact of Severe Acute Respiratory Failure (LUNG SAFE) study. The LUNG SAFE study was an international, prospective study including hypoxemic patients in 459 ICUs from 50 countries across 5 continents.

**Results:**

Of 2813 patients with ARDS, 584 (20.8%) were immunocompromised, 38.9% of whom had an unspecified cause. Pneumonia, nonpulmonary sepsis, and noncardiogenic shock were their most common risk factors for ARDS. Hospital mortality was higher in immunocompromised than in immunocompetent patients (52.4% vs 36.2%; *p* < 0.0001), despite similar severity of ARDS. Decisions regarding limiting life-sustaining measures were significantly more frequent in immunocompromised patients (27.1% vs 18.6%; *p* < 0.0001). Use of noninvasive ventilation (NIV) as first-line treatment was higher in immunocompromised patients (20.9% vs 15.9%; *p* = 0.0048), and immunodeficiency remained independently associated with the use of NIV after adjustment for confounders. Forty-eight percent of the patients treated with NIV were intubated, and their mortality was not different from that of the patients invasively ventilated *ab initio*.

**Conclusions:**

Immunosuppression is frequent in patients with ARDS, and infections are the main risk factors for ARDS in these immunocompromised patients. Their management differs from that of immunocompetent patients, particularly the greater use of NIV as first-line ventilation strategy. Compared with immunocompetent subjects, they have higher mortality regardless of ARDS severity as well as a higher frequency of limitation of life-sustaining measures. Nonetheless, nearly half of these patients survive to hospital discharge.

**Trial registration:**

ClinicalTrials.gov, NCT02010073. Registered on 12 December 2013.

**Electronic supplementary material:**

The online version of this article (10.1186/s13054-018-2079-9) contains supplementary material, which is available to authorized users.

## Background

In recent decades, significant advances in the management of immunocompromised patients have led to improved survival rates [1–3]. Hence, intensive care unit (ICU) admission and invasive life-sustaining treatments are offered with increasing frequency to these patients [3, 4]. However, several studies show that the prognosis of critically ill patients with active malignancies or immunodeficiency remains poor, especially when the cause of ICU admission is acute respiratory distress syndrome (ARDS) requiring invasive mechanical ventilation (IMV) [5–11]. Data about incidence, causes, management, and outcomes of ARDS in immunocompromised patients are scarce. The best ventilatory strategy in this population is still uncertain, and available literature data on the role of noninvasive ventilation (NIV) are conflicting [12–22]. Recently, researchers in the Large Observational Study to Understand the Global Impact of Severe Acute Respiratory Failure (LUNG SAFE study) investigated the incidence, management, and clinical outcomes in patients with acute hypoxemic respiratory failure (AHRF) requiring ventilatory support, with a specific focus on ARDS [23]. This aim of this post hoc subgroup analysis was to describe the epidemiology, clinical characteristics, ventilatory management (with particular attention to the use of NIV), and outcomes of ARDS in the subset of patients with clinically significant immunodeficiency.

## Methods

### LUNG SAFE: patients, study design, and data collection

LUNG-SAFE was an international, multicenter, prospective observational cohort study conducted in a 459 ICUs worldwide. During 4 consecutive weeks in the winter of 2014 (February–March 2014 in the Northern Hemisphere and June–August 2014 in the Southern Hemisphere), participating ICUs enrolled patients undergoing IMV or NIV. Participating ICUs obtained ethics committee approval and either patient consent or waiver of consent as per local guidelines. National coordinators, site investigators, and endorsing societies are listed in Additional file 1. Exclusion criteria were age < 16 years or lack of informed consent when required. Patients were screened daily for AHRF, defined as follows: (1) ratio of partial pressure of arterial oxygen to fraction of inspired oxygen (PaO_2_/FiO_2_) ≤ 300 mmHg while receiving IMV or NIV with positive end-expiratory pressure (PEEP) ≥ 5 cmH_2_O and (2) new radiological pulmonary parenchymal abnormalities. In patients with AHRF, a more detailed set of data was collected to determine whether they met the Berlin definition criteria for ARDS. Data on comorbidities, etiology of AHRF, and risk factors for ARDS were recorded. Data on arterial blood gases, ventilatory support, use of adjunctive therapies (e.g., prone positioning, extracorporeal membrane oxygenation, neuromuscular blockade), severity of ARDS, and other organ involvement by modified nonpulmonary Sequential Organ Failure Assessment (SOFA) score [24] were collected on selected days. The following clinical endpoints were assessed: ICU and hospital survival, censored at 90 days after enrollment; duration of mechanical ventilation; changes in ARDS severity; and decision to withhold or withdraw life-sustaining therapies. A full description of the methods of the LUNG SAFE study, including the full study protocol, case report form (CRF), sample size, and quality control, can be found in the original study paper [23].

### Immunocompromised patient cohort and definitions

We defined “immunocompromised” patients as all patients with at least one of the following conditions listed in the LUNG SAFE CRF: (1) immunosuppression (defined as viral immunosuppression, neoplastic disease, immunosuppressive drugs including steroids, chemotherapy, or congenital immunosuppression), (2) active hematologic malignancy (i.e., still requiring treatment), and (3) active neoplasm (i.e., a neoplasm that has not been resected, still requires treatment, or with metastasis). Patients without these conditions were classified as “controls.” For the purposes of this analysis, the study population was restricted to the subset of patients fulfilling ARDS criteria on day 1 or 2 following the onset of AHRF.

In regard to management, patients were subdivided in three ventilation subgroups: (1) IMV, defined as patients invasively ventilated from day 1, independently of the type of support received after the eventual extubation; (2) NIV, defined as patients treated exclusively with NIV from day 1 to study exit (i.e., ICU discharge or death); and (3) NIV failure, defined as patients initially treated with NIV and subsequently intubated during the study period. The term *NIV* encompassed all forms of NIV modes and interfaces (including continuous positive airway pressure). ARDS severity was assessed from the first to the second day from ARDS onset, according to the Berlin definition criteria: mild (PaO_2_/FiO_2_ 201–300 mmHg), moderate (PaO_2_/FiO_2_ 101–200 mmHg), and severe (PaO_2_/FiO_2_ ≤ 100 mmHg). Changes in ARDS severity were evaluated in patients staying in the ICU for at least 2 days, and they were classified into four categories: (1) no change, (2) worsening (shift to a more severe category), (3) improvement (shift to a less severe category), and (4) resolution. Duration of invasive ventilation was computed as the number of days that the patient required IMV up to day 28. Survival was evaluated at ICU and hospital discharge or at day 90, whichever event occurred first.

### Statistical analysis

Continuous variables were expressed as mean (SD) or median (IQR), and categorical variables were presented as count and percent. No assumptions were made for missing data, which were rare [23]. To assess differences between groups, we used Student’s *t* test or the Wilcoxon rank-sum test (according normality distribution of data) for continuous variables and the χ^2^ or Fisher’s exact test (according sample size) for proportions. We used analysis of variance or the Kruskal-Wallis test (as appropriate) and the χ^2^ test (or Fisher’s exact test) to assess differences among the NIV, NIV failure, and IMV groups. The Bonferroni correction was applied to determine significance in the setting of multiple comparisons.

To evaluate factors associated with the use of NIV, we applied a multivariable logistic regression model, and the independent predictors (demographic characteristics, comorbidities, ARDS risk factors, and clinical parameters concerning the illness severity of ARDS onset) were identified through a stepwise regression approach. This approach combines forward and backward selection methods (combined with a significance level of 0.05 for both entry and retention) in an iterative procedure to select predictors in the final multivariable model. This approach was also applied to identify factors associated with hospital mortality in immunocompromised patients. In this case, the stepwise approach also evaluated as possible predictors ventilator setting variables measured at ARDS onset.

Survival analysis was performed according to the Kaplan-Meier method. We assumed that patients discharged alive from the hospital before 90 days were alive on day 90. The log-rank test was used to compare survival curves among groups.

All *p* values were two-sided, and values less than 0.05 were considered significant. Statistical analyses were carried out with R version 3.3.3 (R Project for Statistical Computing; https://www.r-project.org/) and SAS version 9.4 software (SAS Institute, Cary, NC, USA).

## Results

### Baseline patient characteristics

**Fig. 1:**
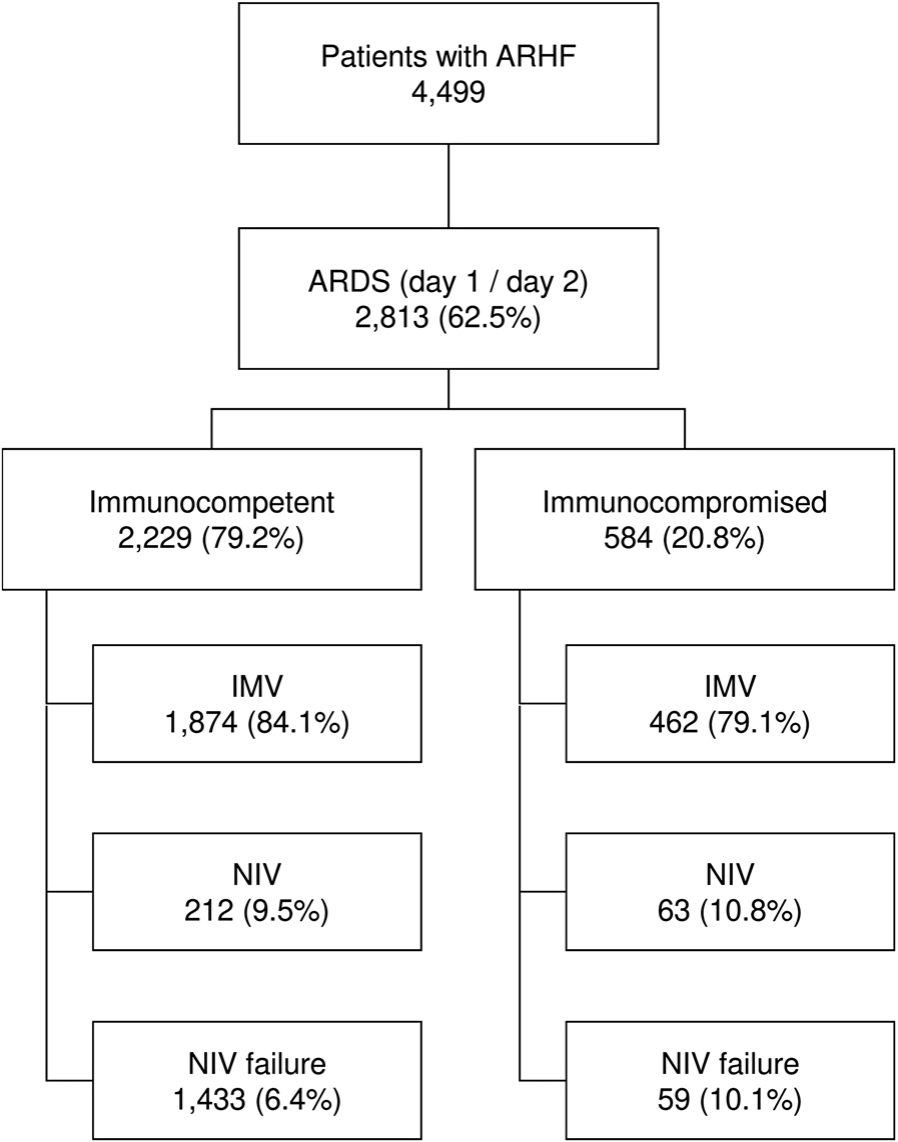
Flow diagram of the study. Flow diagram showing the distribution of patients included in this analysis dataset, according to ventilation subgroup. *AHRF* acute hypoxemic respiratory failure, *ARDS* Acute respiratory distress syndrome, *IMV* Patients invasively ventilated from day 1, independently of the type of support received after the eventual extubation, *NIV* Patients treated exclusively with noninvasive ventilation, from day 1 to study exit, independently of outcome, *NIV failure* Patients initially treated with noninvasive ventilation and subsequently intubated during the study period

A total of 459 ICUs from 50 countries enrolled patients in the LUNG SAFE study. Among 12,906 mechanically ventilated patients, 4499 had AHRF, and of these, 2813 fulfilled the Berlin criteria for ARDS on day 1 or 2. Among ARDS patients, 584 (20.8%) were immunocompromised (Fig. 1). Of these, 232 (39.7%) had an active neoplasm and 138 (23.6%) had a hematologic malignancy, whereas the causes of immunosuppression were not specified in 38.7%. Table 1 shows baseline characteristics of immunocompromised and control patients. Immunocompromised subjects were younger than controls (60.3 vs 61.6 years; *p* = 0.0163) and had a lower body mass index (BMI) (25.5 ± 5.8 vs 28.1 ± 9.2 kg/m^2^; *p* < 0.0001). They had a lower prevalence of chronic obstructive pulmonary disease, diabetes mellitus, and heart failure (New York Heart Association classes III–IV) and a higher incidence of pneumonia, pulmonary vasculitis, and noncardiogenic shock. Among immunocompromised patients, 28.6% had mild, 46.4% moderate, and 25.0% severe ARDS, and the most common risk factors for ARDS were pneumonia (70.5%), nonpulmonary sepsis (16.1%), and noncardiogenic shock (10.3%). Nonpulmonary SOFA score at day 1 and ARDS severity were similar between immunocompromised and controls. Additional file 2 compares comorbidities, ARDS severity, and nonpulmonary SOFA score in the three ventilation subgroups (IMV, NIV, and NIV failure) among immunocompromised patients. Mean patient age in the NIV subgroup was older than in the other two subgroups, and the difference between NIV and IMV was statistically significant (65.2 vs 59.7 years; *p* = 0.0045). Comorbidities and PaO_2_/FiO_2_ were not different among the subgroups. There was a marked difference in the mean nonpulmonary SOFA score, which was significantly higher in IMV than in NIV (7.0 ± 3.9 vs 3.7 ± 3.1; *p* < 0.0001) and NIV failure subgroups (7.0 ± 3.9 vs 5.3 ± 3.6; *p* = 0.0023).

**Table 1:** Patients characteristics in immunocompetent (Control) and immunocompromised (Study) groups

Patients characteristics	Control (*n* = 2229)	Study (*n* = 584)	*p* Value
Sex, age, and BMI			
Women, *n* (%)	837 (37.6)	247 (42.3)	**0.0360**
Age, yr, mean ± SD	61.6 ± 17.1	60.3 ± 15.5	**0.0163**
BMI (kg/m^2^), mean ± SD	28.1 ± 9.2	25.5 ± 5.8	**< 0.0001**
Comorbidities, *n* (%)			
COPD	522 (23.4)	85 (14.6)	**< 0.0001**
Diabetes mellitus	523 (23.5)	90 (15.4)	**< 0.0001**
Hearth failure (NYHA classes III-IV)	260 (11.7)	30 (5.1)	**< 0.0001**
Chronic renal failure	222 (10.0)	64 (11.0)	0.4769
Chronic liver failure (Child-Pugh class C)	91 (4.1)	21 (3.6)	0.5924
Home ventilation	52 (2.3)	7 (1.2)	0.0886
ARDS risk factors, *n* (%)			
Pneumonia	1271 (57.0)	412 (70.5)	**< 0.0001**
Pulmonary contusion	86 (3.9)	1 (0.2)	**< 0.0001**
Pulmonary vasculitis	7 (0.3)	7 (1.2)	**0.0140**
Major trauma	111 (5.0)	1 (0.2)	**< 0.0001**
Aspiration of gastric contents	348 (15.6)	54 (9.2)	**< 0.0001**
Pancreatitis	54 (2.4)	5 (0.9)	**0.0187**
Noncardiogenic shock	154 (6.9)	60 (10.3)	**0.0063**
Drug overdose	46 (2.1)	5 (0.9)	0.0515
Severe burns	8 (0.4)	0 (0.0)	0.2183
Inhalational injury	58 (2.6)	12 (2.1)	0.4498
Drowning	1 (0.04)	1 (0.2)	0.3722
Nonpulmonary sepsis	361 (16.2)	94 (16.1)	0.9535
Blood transfusions	82 (3.7)	29 (5.0)	0.1550
Other risk factors	61 (2.7)	12 (2.2)	0.4925
None	194 (8.7)	40 (6.8)	0.1487
Cause of immunosuppression, *n* (%)			
Known (hematologic and/or active neoplasm)	–	357 (61.1)	–
Unknown	–	227 (38.9)	–
Illness severity at ARDS onset			
Nonpulmonary SOFA score^a^, mean ± SD			
First day of ARDS	6.2 ± 4.1	6.5 ± 4.0	0.1150
Second day of ARDS	6.3 ± 4.3	6.7 ± 4.3	**0.0283**
PaO_2_/FiO_2_ ratio, mmHg, mean ± SD	161.3 ± 67.1	157.2 ± 67.9	0.1660
Mild ARDS^b^, *n* (%)	666 (29.9)	167 (28.6)	0.5455
Moderate ARDS^b^, *n* (%)	1067 (47.9)	271 (46.4)	0.5280
Severe ARDS^b^, *n* (%)	496 (22.3)	146 (25.0)	0.1590

*Abbreviations: BMI* Body mass index, *ARDS* Acute respiratory distress syndrome, *COPD* Chronic obstructive pulmonary disease, *NYHA* New York Heart Association, *SOFA* Sequential Organ Failure Assessment, *PaO*_*2*_*/FiO*_*2*_ Ratio of partial pressure of arterial oxygen to fraction of inspired oxygen

*Note:* Bold *p* values represent a statistically significant difference between the two groups

^a^Nonpulmonary SOFA score adjusted for missing values

^b^Severity of ARDS was evaluated according to the Berlin definition

### Type of ventilatory support, ventilator setting, and adjunctive measure/therapies

Figure 1 summarizes the type of ventilatory support in enrolled patients. On day 1 of ARDS, IMV was the most frequent type of ventilatory approach in both groups; however, NIV use as first-line treatment was significantly more frequent in immunocompromised than in immunocompetent patients (20.9% vs 15.9%; *p* = 0.0044). The proportion of patients remaining on NIV from day 1 to study exit (NIV subgroup) was similar, whereas the incidence of NIV failure was significantly higher in immunocompromised patients (10.1% vs 6.4%; *p* = 0.0021).

A multivariable logistic regression model revealed that, adjusting on confounders, immunodeficiency was independently associated with the use of NIV (OR, 1.567; 95% CI, 1.217–2.017; *p* = 0.0005). Other factors associated with NIV are shown in Additional file 3.

Additional file 4 compares the ventilator settings on the first day of ARDS between immunocompromised and control patients: FiO_2_ and respiratory rate and PEEP were statistically significantly higher in immunocompromised patients, but the difference for PEEP was not clinically relevant. There was no difference in tidal volume, peak and plateau pressures, and the proportion of patients with spontaneous ventilation. No significant differences were observed in adjunctive therapies, except for a significantly higher use of continuous neuromuscular blocking agents in the immunocompromised group (22.6% vs 18.5%; *p* = 0.0266) (Additional file 5). Additional file 6 describes ventilator settings in immunocompromised and immunocompetent (control) patients, stratified by the type of ventilator support (IMV, NIV, NIV failure).

### Clinical endpoints

**Fig. 2:**
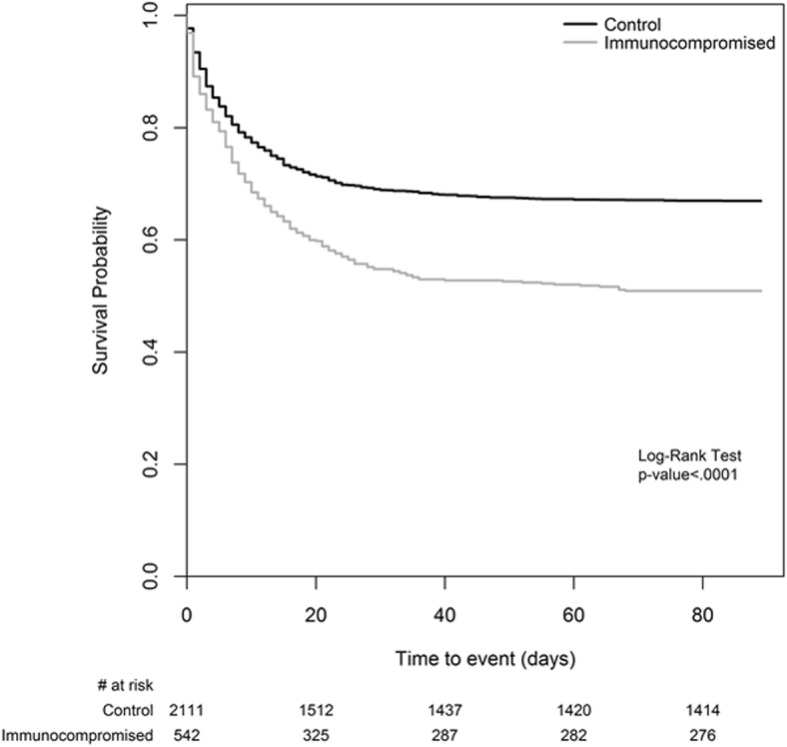
Kaplan-Meier curve for hospital survival. Mortality was defined as mortality at hospital discharge or at 90 days after onset of acute hypoxemic respiratory failure, whichever event occurred first. We assumed that patients discharged alive from the hospital before 90 days were alive on day 90. *Note:* The number of patients at risk reported at the bottom of the figure is referred to as the end of the corresponding day

Table 2 compares selected clinical endpoints in immunocompromised and control patients. Hospital mortality and ICU mortality were significantly higher in immunocompromised subjects (respectively, 52.4% vs 36.2%, *p* < 0.0001; and 45.5% vs 31.3, *p* < 0.0001), whereas there was no difference in duration of mechanical ventilation and changes in ARDS severity. Survival curves for hospital (or 90-day) mortality are shown in Fig. 2. The decision to withhold and/or withdraw life-sustaining measures was significantly more frequent in immunocompromised patients (Table 2). The same clinical endpoints were also analyzed in the cohort of immunocompromised patients according to the ventilation subgroup (Table 3). Duration of mechanical ventilation and decisions of limitation (both withholding and withdrawal) of life-sustaining measures were not different among the subgroups. ICU mortality was significantly lower in NIV patients than in the IMV (28.6% vs 46.3%; *p* = 0.0078) and NIV failure (28.6% vs 57.6%; *p* = 0.0012) subgroups. Of the NIV patients who died, 68% had a limitation of life-sustaining measures. The incidence of NIV failure was 48%. ICU and hospital mortality of patients with NIV failure were significantly higher than those of patients managed exclusively with NIV (respectively, 57.6% vs 28.6%, *p* = 0.012; and 62.7 vs 39.7%, *p* = 0.011), whereas they did not differ from those of IMV patients. Survival curves for hospital (or 90-day) mortality of immunocompromised patients stratified by ARDS severity and by ventilation subgroups are shown in Additional files 7 and 8, respectively. In a multivariable logistic regression model, factors independently associated with hospital mortality in immunocompromised patients were higher nonpulmonary SOFA score (OR, 1.079; 95% CI, 1.026–1.134; *p* = 0.0032), higher peak inspiratory pressure level (OR, 1.028; 95% CI, 1.007–1.051; *p* = 0.0097), lower PaO_2_/FiO_2_ ratio (OR, 0.995; 95% CI, 0.992–0.998; *p* = 0.0022), lower degree of improvement in PaO_2_/FiO_2_ ratio between day 1 and day 2 of ARDS (OR, 0.996; 95% CI, 0.993–0.999; *p* = 0.0058), and lower BMI (OR, 0.944; 95% CI, 0.91–0.98; *p* = 0.0023) (Additional file 9). According to the investigators’ clinical judgment, in immunocompromised patients, the most common main factor leading to death in ICU was respiratory failure (51.5%), followed by cardiovascular failure. In contrast, cardiovascular failure was the most common factor in the control group (Additional file 10).

**Table 2:** Clinical endpoints in immunocompetent (Control) and immunocompromised (Study) patients

Clinical endpoints	Control (*n* = 2229)	Study (*n* = 584)	*p* Value
IMV during ICU stay, *n* (%)	1874 (84.1)	462 (79.1)	**0.0044**
NIV success during ICU stay, *n* (%)	212 (9.5)	63 (10.8)	0.3551
NIV failure during ICU stay, *n* (%)	143 (6.4)	59 (10.1)	**0.0021**
Duration of mechanical ventilation, d, median (Q_1_–Q_3_)	8.0 (4.0–15.0)	8.0 (4.0–14.0)	0.4213
Progression/regression of ARDS^a^, *n* (%)			0.5613
No change	824 (41.7)	201 (39.6)	
Progression	214 (10.8)	55 (10.8)	
Regression	422 (21.3)	123 (24.2)	
Resolution	518 (26.2)	129 (25.4)	
Limitation of life-sustaining measures, *n* (%)			
Decision to withhold life-sustaining measures	415 (18.6)	158 (27.1)	**< 0.0001**
Decision to withdraw life-sustaining measures	356 (16.0)	129 (22.1)	**0.0005**
Decision to withhold or withdraw life-sustaining measures	507 (22.7)	195 (33.4)	**< 0.0001**
ICU mortality^b^, *n* (%)	698 (31.3)	266 (45.5)	**< 0.0001**
Hospital mortality^c^, *n* (%)			
All patients	804 (36.2)	304 (52.4)	**< 0.0001**
Patients with limitations of life-sustaining measures^d^	419 (82.6)	173 (88.7)	**0.0473**

*Abbreviations: ARDS* Acute respiratory distress syndrome, *IMV* Invasive mechanical ventilation, *ICU* Intensive care unit, *NIV* Noninvasive mechanical ventilation, *Q*_*1*_ First quartile, *Q*_*3*_ Third quartile

^a^Change in ARDS severity (according Berlin definition) was not evaluable for 327 pients (251 immunocompetent and 76 immunocompromised patients)

^b^Mortality is defined as mortality at ICU discharge or at the 90th day in the ICU after onset of acute hypoxemic respiratory failure, whichever event occurred first

^c^Mortality is defined as mortality at hospital discharge or at the 90th day in the hospital after onset of acute hypoxemic respiratory failure, whichever event occurred first

^d^Mortality assessed on patients with a decision to withhold or withdraw life-sustaining measures

*Note:* Bold *p* values represent a statistically significant difference between the two groups

**Table 3:** Clinical endpoints in immunocompromised (Study) patients according to ventilation subgroup

Clinical endpoints	IMV (*n* = 462)	NIV (*n* = 63)	NIV failure (*n* = 59)	*p* Value
Duration of mechanical ventilation, d, median (Q_1_–Q_3_)	8.0 (4.0–14.0)	–	8.0 (5.0–15.0)	0.4352
Progression/regression of ARDS^a^, *n* (%)				
No change	161 (40.1)	18 (32.1)	22 (43.1)	0.4449
Progression	43 (10.7)	5 (8.9)	7 (13.7)	0.7199
Regression	103 (25.7)	4 (7.1)^b^	16 (31.4)^c^	**0.0045**
Resolution	94 (23.44)	29 (51.8)^b^	6 (11.8)^c^	**< 0.0001**
Limitation of life-sustaining measures, *n* (%)				
Decision to withhold life-sustaining measures	124 (26.8)	18 (28.6)	16 (27.1)	0.9587
Decision to withdraw life-sustaining measures	101 (21.9)	14 (22.2)	14 (23.7)	0.9480
Decision to withhold or withdraw life-sustaining measures	154 (33.3)	21 (33.3)	20 (33.9)	0.9962
Before IMV or NIV start	1 (0.6)	0 (0.0)	1 (5.0)	0.2062
ICU mortality^d^, *n* (%)	214 (46.3)	18 (28.6)^b^	34 (57.6)^c^	**0.0043**
Hospital mortality^e^, *n* (%)				
All patients	242 (52.8)	25 (39.7)	37 (62.7)^c^	**0.0362**
Patients with limitations of life-sustaining measures^f^	137 (89.0)	17 (81.0)	19 (95.0)	0.3803

*Abbreviations: ARDS* Acute respiratory distress syndrome, *IMV* Patients invasively ventilated from day 1, independently of the type of support received after the eventual extubation, *NIV* Patients treated exclusively with noninvasive ventilation, from day 1 to study exit, independently of outcome, *NIV failure* Patients initially treated with noninvasive ventilation and subsequently intubated during the study period, *Q*_*1*_ First quartile, *Q*_*3*_ Third quartile

^a^Change in ARDS severity (according Berlin definition) was not evaluable for 76 immunocompromised patients (61 IMV, 7 NIV, and 8 NIV failure)

^b^Statistically significant different from the IMV group

^c^Statistically significant different from the NIV group

^d^Mortality is defined as mortality at ICU discharge or at the 90th day in ICU after onset of acute hypoxemic respiratory failure, whichever event occurred first

^e^Mortality is defined as mortality at hospital discharge or at the 90th day in hospital, after onset of acute hypoxemic respiratory failure, whichever event occurred first

^f^Mortality assessed in patients with a decision to withhold or withdraw life-sustaining measures

*Note:* Bold *p* values represent a statistically significant difference among the three groups

Additional file 11 describes patient characteristics and clinical endpoints in immunocompetent (control) patients according to type of ventilator support. Because the lack of a precise definition of the cause of immunosuppression in a relevant proportion of patients may affect the strength of our findings, we compared baseline patient characteristics and clinical outcomes in patients with a “known” cause of immunosuppression (i.e., those with active hematologic malignancy or active neoplasm) and in patients with an unspecified (“unknown”) cause of immunosuppression (i.e., those indicated in the CRF with the generic term *immunosuppression*). The results of this analysis are reported in Additional file 12. Briefly, patients with an unspecified cause of immunosuppression were significantly younger (55.7 ± 15.6 vs 63.1 ± 14.8 years; *p* < 0.0001) and had lower hospital mortality (41.6% vs 59.3%; *p* < 0.0001) and fewer limitations of life-sustaining measures (27.3% vs 37.3%; *p* = 0.013).

## Discussion

The main findings of this analysis can be summarized as follows: immunosuppression is frequent in ARDS patients; causes of ARDS in immunocompromised patients are mainly related to infection; immunocompromised patients are more likely to receive NIV as first-line ventilatory treatment; and the outcome of ARDS is worse and limitation of life-sustaining measures is more frequent in immunocompromised patients. Among the 2813 ARDS patients included in the LUNG SAFE study, one-fifth were immunocompromised, and 62.7% of them had an active malignancy. In line with recent literature [18, 21], a diagnosis of active malignancy is considered as a cause of immunosuppression, owing to the negative effects on immune function of anticancer treatments and of the malignancy itself. All other causes of immune deficiency were identified by the generic variable “immunosuppression.” To the best of our knowledge, this was the first prospective, multicenter study conducted on a large cohort of immunocompromised patients with a diagnosis of ARDS according to Berlin definition criteria [25], whereas previous studies relied on the definition of the American-European Consensus Conference on ARDS [26]. As expected, ARDS in immunocompromised patients was associated mainly with an infectious cause. Pneumonia, noncardiogenic shock, and pulmonary vasculitis were significantly more frequent in immunocompromised patients, whereas other risk factors for ARDS were more frequently represented in the control group.

Immunocompromised subjects had a significantly higher ICU and hospital mortality, despite similar ARDS severity and nonpulmonary SOFA score. Another subanalysis of the same database confirmed that active neoplasm, hematologic malignancies, and immunosuppression are independently associated with increased mortality [27]. In addition, the higher frequency of limitation of life-sustaining measures (probably as a result of perceived futility) may have contributed to the increased mortality of immunocompromised patients [27]. This is in line with the results of Laffey et al., who found that immunosuppression and cancer were among the factors associated with increased likelihood of limitation of life-sustaining therapies [27].

Hospital mortality of our patients was lower than that (64%) reported by Azoulay et al. in a large retrospective analysis of 1004 patients with cancer and ARDS admitted to the ICU over a period of 21 years [28]. In that study, mortality did drop from 89% in the 1990–1995 period to 52% in the 2006–2011 period, matching the mortality rate of our population.

The actual advantage of ICU admission of immunocompromised patients remains debated [3, 4, 10, 29, 30]. Our results demonstrate that almost 50% of immunocompromised patients with ARDS survive to hospital discharge, and this may support the decision to offer them at least an “ICU trial” [31]. Interestingly, our data show that once immunocompromised patients are intubated and invasively ventilated, they are managed very similarly to the general ARDS population with regard to ventilator settings and use of adjunctive therapies. As an example, the use of advanced “rescue” treatments (such as prone positioning and even extracorporeal membrane oxygenation) was similar between immunocompromised and control patients.

Two important questions on the optimal ventilatory management of AHRF in immunocompromised patients remain unanswered. First, is NIV the optimal first-line ventilatory support? Two randomized controlled trials conducted almost 20 years ago showed that NIV, compared with standard oxygen therapy, significantly reduces the rate of intubation and mortality [16, 17], but these findings have not been confirmed in more recent studies. A recent randomized trial on 374 immunocompromised patients with AHRF did not find any benefit of NIV over standard oxygen therapy [18]. Similarly, a post hoc analysis on immunocompromised patients enrolled in a large randomized trial comparing different noninvasive oxygenation strategies showed that first-line NIV was associated with the highest risk of intubation and mortality compared with standard oxygen and high-flow nasal cannula (HFNC) oxygen [21]. In our study, 20.9% of immunocompromised patients received NIV as first-line ventilatory approach compared with 15.9% of controls, and the multivariable analysis revealed that immunodeficiency was independently associated with the use of NIV. This frequency of use of NIV equals exactly that reported by Gristina in a large population of patients with hematologic malignancies admitted to ICU in the years 2002–2006 [26]. In Azoulay’s study, the global rate of NIV application was 38.6%, but it decreased over the years, dropping to 26% in the period 2006–2011 [28]. In our study, patients treated exclusively with NIV had significantly lower mortality than patients requiring invasive ventilation. Of note, the majority of NIV patients who died had a decision of limitation of life-sustaining measures. Importantly, whereas ARDS severity was not different among ventilation subgroups, NIV patients had a markedly lower nonpulmonary SOFA score (Additional file 2: Table S1), indicating that patients with more severe organ failures were more frequently treated with invasive ventilatory support. However, the multivariable regression analysis did not identify the need for invasive ventilation as an independent predictor of death. This represents a major difference with Azoulay’s study, where IMV (especially after failure of NIV) was a strong predictor of poor outcome [28]. Again, this can be explained at least in part by considering that Azoulay’s study included patients from the year 1990, when the techniques of mechanical ventilation were completely different and protective ventilation was certainly not the standard of care.

The impact of NIV failure on patients’ outcomes is the second important issue. We observed a significantly higher incidence of NIV failure in immunocompromised patients than in controls, in keeping with the observation of Thille that a diagnosis of active cancer is independently associated with NIV failure [32]. In our study, in 48% of immunocompromised patients initially treated with NIV, NIV failed, and they had a significantly worse mortality than patients successfully managed with NIV, as previously reported by Bellani et al. [23]. Less expected was the finding that mortality of NIV failure patients was not different from that of the patients managed *ab initio* with IMV. However, two factors may limit the relevance of this observation: the relatively low number of patients in the NIV failure subgroup and the lack of information on the actual duration (i.e., in hours rather than days) of the NIV period before intubation, which would be important to know in the light of literature data showing that delaying endotracheal intubation after a prolonged NIV trial may negatively impact patient survival [19, 20]. In line with our data, Demoule et al. recently observed a progressive reduction of the impact of NIV failure on mortality in a large population of AHRF patients also including immunocompromised subjects [33]. Taken together, these data probably suggest that better patient selection, earlier recognition of failure, and improvement in ventilation techniques may have contributed to limit the impact of NIV failure on mortality in recent years.

### Limitations

The present study has several limitations. First, it is a post hoc analysis of a prospective multicenter observational trial, and unknown confounders associated with the subgroup analysis may bias the results. Although the data were prospectively collected from a high number of centers from 50 countries, different approaches to clinical decisions from different centers (e.g., decisions on withholding or withdrawing of life-sustaining measures) may have influenced the outcomes. Second, the criteria used to define the immunocompromised cohort were quite heterogeneous. It was impossible to stratify the patients according to the prognosis of baseline disease and to the severity of immune deficiency. Indeed, causes of immunosuppression, other than malignancies, were not specified in nearly 40% of the whole cohort of immunocompromised patients. Moreover, in patients with cancer, no information was available on the type of cancer, its staging, and the nature and timing of anticancer treatments. This lack of information, related to the LUNG SAFE original CRF, should be considered a major limitation because the outcome of immunocompromised patients is strictly dependent on the type of underlying diseases and associated therapeutic approach. All these factors may limit the generalizability of our findings. Third, to limit the burden on investigators, data were collected once daily only, and information on the actual hours of duration of ventilatory treatments was not available. This is particularly relevant for patients treated with NIV because the precise duration of NIV before the eventual intubation might be important to understand the impact of NIV failure on outcome [24]. Fourth, no information were provided on the type of interface used for NIV, a factor that can affect the outcome of NIV [34]. Moreover, patients treated with HFNC oxygen therapy were excluded from the LUNG SAFE study because they did not fulfill criteria for ARDS. Fifth, the LUNG SAFE study was conducted in a very large number of ICUs with different experience in the treatment of ARDS. This may be particularly relevant for immunocompromised patients, who may have better outcomes if treated in highly experienced, dedicated units [35].

## Conclusions

Immunocompromised patients represent an important proportion of ARDS patients in the ICU. Compared with immunocompetent subjects, they had higher mortality, regardless of ARDS severity, and a higher frequency of limitation of life-sustaining measures. Nonetheless, nearly half of these patients survive to hospital discharge. They were more likely to receive NIV as the first ventilator strategy, and those who did not require invasive ventilation had a lower mortality. Mortality of immunocompromised patients who failed NIV was not different from that of patients treated *ab initio* with IMV. These data should be considered in light of the nonspecific criteria used to define the immunocompromised population and the potentially heterogeneous approaches to clinical decision making in the participating centers.

## Additional files

Additional file 1: List of LUNG SAFE investigators. Names and affiliations of the LUNG SAFE investigators. (PDF 172 kb)

Additional file 2: **Table S1.** Patient characteristics of immunocompromised patients according to the type of ventilator support. This table shows patient characteristics, including comorbidities, ARDS risk factors, and illness severity at ARDS onset of immunocompromised patients according to the type of ventilator support. (PDF 74 kb)

Additional file 3: **Table S2.** Factors associated with the use of noninvasive ventilation. Multivariate logistic regression model describing the factors associated with the use of noninvasive ventilation. (PDF 49 kb)

Additional file 4: **Table S3.** Ventilator settings during the first day of ARDS in the immunocompetent (Control) and immunocompromised (Study) groups. This table shows ventilator settings during the first day of ARDS in the immunocompetent (Control) and immunocompromised (Study) groups. (PDF 50 kb)

Additional file 5: **Table S4.** Adjunctive measures/therapies during at least one day during follow-up in immunocompetent and immunocompromised patients. This table shows the proportions of adjunctive measures/therapies during at least one day during follow-up in immunocompetent and immunocompromised patients. (PDF 97 kb)

Additional file 6: **Table S6.** Ventilator settings during the first day of ARDS in immunocompetent (Control) and immunocompromised (Study) patients, stratified by the type of ventilatory support (IMV, NIV, NIV failure). (PDF 60 kb)

Additional file 7: **Figure S1.** Kaplan-Meier curve for hospital survival in immunocompromised patients according to ARDS severity. Kaplan-Meier curve for hospital survival in immunocompromised patients according to ARDS severity. Mortality is defined as mortality at hospital discharge or at 90 days after onset of acute hypoxemic respiratory failure, whichever event occurred first. We assumed that patients discharged alive from the hospital before 90 days were alive on day 90. Severity of ARDS was evaluated at the day of onset according to the Berlin definition. *Note:* The number of patients reported in the bottom of figure is referred to as the end of the corresponding day. (PDF 402 kb)

Additional file 8: **Figure S2.** This figure shows a Kaplan-Meier curve for hospital survival of immunocompromised patients according to the ventilation subgroup. This figure shows a Kaplan-Meier curve for hospital survival of immunocompromised patients according to the ventilation subgroup. Mortality is defined as mortality at hospital discharge or at 90 days after onset of acute hypoxemic respiratory failure, whichever event occurred first. We assumed that patients discharged alive from the hospital before 90 days were alive on day 90. Type of ventilator support: *IMV* Patients invasively ventilated from day 1, independently of the type of support received after the eventual extubation; *NIV* Patients treated exclusively with noninvasive ventilation, from day 1 to study exit, independently of outcome; *NIV failure* Patients initially treated with noninvasive ventilation and subsequently intubated during the study period. *Note:* The number of patients reported in the bottom of the figure is referred to as the end of the corresponding day. (PDF 396 kb)

Additional file 9: **Table S5.** Factors associated with hospital mortality in immunocompromised patients. Multivariate logistic regression model describing the factors associated with hospital mortality in immunocompromised patients. (PDF 49 kb)

Additional file 10: **Table S9.** The most important factors leading to death in the ICU in immunocompetent and immunocompromised patients. (PDF 44 kb)

Additional file 11: **Table S7.** Patient characteristics and clinical endpoints of immunocompetent patients, according to the type of ventilatory support. (PDF 88 kb)

Additional file 12: **Table S8.** Patients’ characteristics and clinical endpoints of immunocompromised (study) patients, according to the cause of immunosuppression (known, unknown). (PDF 79 kb)
