# Supplementary material for: Immunocompromised patients with acute respiratory distress syndrome: secondary analysis of the LUNG SAFE database
Source: Crit Care. 2018 Jun 12;22:157. doi: 10.1186/s13054-018-2079-9 (PMC5998562; doi:10.1186/s13054-018-2079-9)
Supplement: Supplementary file 2 — Table S1. Patient characteristics of immunocompromised patients according to the type of ventilator support. This table shows patient characteristics, including comorbidities, ARDS risk factors, and illness severity at ARDS onset of immunocompromised patients according to the type of ventilator support. (PDF 74 kb) [file 13054_2018_2079_MOESM2_ESM.pdf]

Table S1: Patient characteristics of immunocompromised (Study) patients according to the type of ventilatory support

	IMV (n=462)	NIV (n=63)	NIV failure (n=59)	p value
Sex, age and BMI				
Women, n (%)	198 (42.9)	27 (42.9)	22 (37.3)	0.7139
Age (years), mean ± SD	59.7 ± 15.5	65.2 ± 16.5*	59.7 ± 14.3	**0.0158**
BMI (kg/m^2), mean ± SD	25.7 ± 6.0	24.7 ± 5.4	24.3 ± 4.8	0.1140
Co-morbidities, n (%)				
COPD	69 (14.9)	12 (19.0)	4 (6.8)	0.1392
Diabetes mellitus	72 (15.6)	10 (15.9)	8 (13.6)	0.9157
Hearth failure (NYHA classes III-IV)	24 (5.2)	3 (4.8)	3 (5.1)	1.0000
Chronic renal failure	48 (10.4)	10 (15.9)	6 (10.2)	0.4168
Chronic liver failure (Child-Pugh Class C)	19 (4.1)	0 (0.0)	2 (3.4)	0.3044
Home ventilation	5 (1.1)	1 (1.6)	1 (1.7)	0.4482
ARDS risk factors, n (%)				
Pneumonia	311 (67.3)	51 (81.0)	50 (84.7)*	**0.0035**
Pulmonary contusion	1 (0.2)	0 (0.0)	0 (0.0)	1.0000
Pulmonary vasculitis	3 (0.6)	3 (4.8)*	1 (1.7)	**0.0255**
Major trauma	1 (0.2)	0 (0.0)	0 (0.0)	1.0000
Aspiration of gastric contents	50 (10.8)	4 (6.3)	0 (0.0)*	**0.0182**
Pancreatitis	4 (0.9)	0 (0.0)	1 (1.7)	0.4794
Non- cardiogenic shock	54 (11.7)	3 (4.8)	3 (5.1)	0.0906
Drug-overdose	3 (0.6)	1 (1.6)	1 (1.7)	0.2808
Severe burns	0 (0.0)	0 (0.0)	0 (0.0)	-
Inhalational injury	12 (2.6)	0 (0.0)	0 (0.0)	0.3464
Drowning	1 (0.2)	0 (0.0)	0 (0.0)	1.0000
Non-pulmonary sepsis	83 (18.0)	6 (9.5)	5 (8.5)	0.0565
Blood transfusions	27 (5.8)	1 (1.6)	1 (1.7)	0.2436
Other risk factors	12 (2.6)	0 (0.0)	1 (1.7)	0.5942
None	33 (7.1)	4 (6.3)	3 (5.1)	0.9550
Cause of immunosuppression, n (%)				0.8968
Known (hematologic and/or active neoplasm)	283 (61.3)	37 (58.7)	37 (62.7)	
Unknown	179 (38.7)	26 (41.3)	22 (37.3)	
Illness severity at ARDS onset				
Non-pulmonary SOFA score[a], mean ± SD	7.0 ± 3.9	3.7 ± 3.1*	5.3 ± 3.6*	**<0.0001**
PaO_2/FiO_2 ratio (mm Hg), mean ± SD	159.4 ± 70.0	154.5 ± 58.4	142.5 ± 59.2	0.2359
Mild ARDS[b], n (%)	143 (31.0)	15 (23.8)	9 (15.3)*	**0.0286**
Moderate ARDS[b], n (%)	201 (43.5)	37 (58.7)*	33 (55.9)	**0.0228**
Severe ARDS[b], n (%)	118 (25.5)	11 (17.5)	17 (28.8)	0.2953

Abbreviations: ARDS: acute respiratory distress syndrome; COPD: chronic obstructive pulmonary disease; IMV: patients invasively ventilated from Day 1, independently of the type of support received after the eventual extubation; NIV: patients treated exclusively with non-invasive ventilation, from Day 1 to study exit, independently of outcome; NIV failure: patients initially treated with non-invasive ventilation and subsequently intubated during the study period; NYHA: New York Heart Association; SD: standard deviation; SOFA: sequential organ failure assessment.

a. Non pulmonary SOFA score adjusted for missing values

b. Severity of ARDS was evaluated according to the Berlin definition

Note: Bold p values shows a statistically significant difference among the three groups

* Statistically significant different from IMV group; † Statistically significant different from NIV group
