# Supplementary material for: Immunocompromised patients with acute respiratory distress syndrome: secondary analysis of the LUNG SAFE database
Source: Crit Care. 2018 Jun 12;22:157. doi: 10.1186/s13054-018-2079-9 (PMC5998562; doi:10.1186/s13054-018-2079-9)
Supplement: Supplementary file 3 — Table S2. Factors associated with the use of noninvasive ventilation. Multivariate logistic regression model describing the factors associated with the use of noninvasive ventilation. (PDF 49 kb) [file 13054_2018_2079_MOESM3_ESM.pdf]

Table S2: Multivariable logistic regression model: factors associated with the use of non-invasive ventilation (n=2,784).

Effect	OR	95% CI		P value
Age (year)	1.017	1.010	1.024	<.0001
Non-pulmonary SOFA score[a]	0.789	0.762	0.817	<.0001
Immunocompromised (ref. No)	1.567	1.217	2.017	0.0005
P/F ratio (mmHg)	0.998	0.996	1.000	0.0193
Aspiration of gastric contents (ref. No)	0.376	0.250	0.566	<.0001
Non-pulmonary sepsis (ref. No)	0.630	0.444	0.893	0.0095
Inhalation injury (ref. No)	0.211	0.064	0.692	0.0102
Non-cardiogenic shock (ref. No)	0.555	0.311	0.989	0.0457
Other ARDS risk factors (ref. No)	0.248	0.088	0.700	0.0085
Home ventilation (ref. No)	1.977	1.101	3.551	0.0225
Chronic renal failure (ref. No)	1.925	1.382	2.680	0.0001
Chronic liver failure (ref. No)	2.021	1.005	4.066	0.0485

Abbreviation: ARDS: acute respiratory distress syndrome; CI: confidence interval; OR: odd ratio; SOFA: sequential organ failure assessment.

a. Non pulmonary SOFA score adjusted for missing values
