# Supplementary material for: Immunocompromised patients with acute respiratory distress syndrome: secondary analysis of the LUNG SAFE database
Source: Crit Care. 2018 Jun 12;22:157. doi: 10.1186/s13054-018-2079-9 (PMC5998562; doi:10.1186/s13054-018-2079-9)
Supplement: Supplementary file 4 — Table S3. Ventilator settings during the first day of ARDS in the immunocompetent (Control) and immunocompromised (Study) groups. This table shows ventilator settings during the first day of ARDS in the immunocompetent (Control) and immunocompromised (Study) groups. (PDF 50 kb) [file 13054_2018_2079_MOESM4_ESM.pdf]

Table S3: Ventilator settings during the first day of acute respiratory distress syndrome, in immunocompetent (Control) and immunocompromised (Study) patients

	**Control (n=2229)**	**Study (n=584)**	**p value**
FiO_2, mean ± SD	0.6 ± 0.2	0.7 ± 0.2	**0.0019**
Set respiratory rate (bpm), mean ± SD	18.0 ± 9.1	19.0 ± 7.3	**0.0007**
Total respiratory rate (bpm), mean ± SD	21.3 ± 8.9	22.9 ± 7.3	**<0.0001**
Tidal volume (mL/kg IBW), mean ± SD	7.7 ± 2.0	7.6 ± 2.0	0.1374
PEEP (cmH_2O), mean ± SD	8.2 ± 3.2	8.4 ± 3.2	**0.0246**
PIP (cmH_2O), mean ± SD	25.6 ± 8.5	26.0 ± 9.4	0.2854
Plateau pressure (cmH_2O)[a], mean ± SD	23.0 ± 6.1	23.8 ± 6.2	0.1259
Patients with spontaneous ventilation (triggering ventilator)[b], n (%)	1258 (57.4)	342 (59.4)	0.3971

Abbreviations: IBW: ideal body weight; PEEP: positive end-expiratory pressure; PIP: peak inspiratory pressure; SD: standard deviation.

a. Plateau pressure were available for 742 patients (571 immunocompetent and 171 immunocompromised patients)

b. Information was available for 2767 patients (2191 immunocompetent and 576 immunocompromised patients)

Note: Bold p values shows a statistically significant difference between the two groups
