# Supplementary material for: Immunocompromised patients with acute respiratory distress syndrome: secondary analysis of the LUNG SAFE database
Source: Crit Care. 2018 Jun 12;22:157. doi: 10.1186/s13054-018-2079-9 (PMC5998562; doi:10.1186/s13054-018-2079-9)
Supplement: Supplementary file 5 — Table S4. Adjunctive measures/therapies during at least one day during follow-up in immunocompetent and immunocompromised patients. This table shows the proportions of adjunctive measures/therapies during at least one day during follow-up in immunocompetent and immunocompromised patients. (PDF 97 kb) [file 13054_2018_2079_MOESM5_ESM.pdf]

Table S4: Proportion of use of adjunctive measures/therapies during at least one day during follow-up, in immunocompetent (Control) and immunocompromised (Study) groups

	**Control (n=2229)**	**Study (n=584)**	**p value**
Adjunctive measure/therapy, n (%)			
Prone positioning	152 (6.8)	47 (8.0)	0.3026
Recruitment maneuvers	414 (18.6)	121 (20.7)	0.2395
ECMO	63 (2.8)	15 (2.6)	0.7355
Renal replacement therapy	428 (19.2)	122 (20.9)	0.3596
Continuous sedation	1666 (74.7)	455 (77.9)	0.1135
Continuous NMBs	413 (18.5)	132 (22.6)	**0.0266**
Esophageal pressure monitoring	16 (0.7)	3 (0.5)	0.7797
Tracheostomy	250 (11.2)	76 (13.0)	0.2269

Abbreviations: ECMO: extra corporeal membrane oxygenation; NMB: neuromuscular blocking agent.

Note: Bold p values shows a statistically significant difference between the two groups
