# Supplementary material for: Immunocompromised patients with acute respiratory distress syndrome: secondary analysis of the LUNG SAFE database
Source: Crit Care. 2018 Jun 12;22:157. doi: 10.1186/s13054-018-2079-9 (PMC5998562; doi:10.1186/s13054-018-2079-9)
Supplement: Supplementary file 6 — Table S6. Ventilator settings during the first day of ARDS in immunocompetent (Control) and immunocompromised (Study) patients, stratified by the type of ventilatory support (IMV, NIV, NIV failure). (PDF 60 kb) [file 13054_2018_2079_MOESM6_ESM.pdf]

Table S6: Ventilator settings during the first day of acute respiratory distress syndrome, in immunocompetent (Control) and immunocompromised (Study) patients stratified by the type of ventilatory support (IMV, NIV, NIV failure)

	IMV	NIV	NIV failure	P value
**Immunocompetent patients (n=2229)**				
Number of patients	1874	212	143	
FiO_2, median (Q_1-Q_3)	0.6 (0.5-0.8)	0.5 (0.4-0.7)*	0.6 (0.5-0.8)†	**0.0002**
Set respiratory rate (bpm), mean ± SD	18.3 ± 9.2	14.6 ± 8.3*	16.4 ± 7.4	**<0.0001**
Total respiratory rate (bpm), mean ± SD	20.6 ± 9.0	25.3 ± 6.8*	23.8 ± 7.5*	**<0.0001**
Tidal volume (mL/kg IBW), mean ± SD	7.6 ± 1.9	8.2 ± 2.7*	8.4 ± 2.6*	**0.0003**
PEEP (cmH_2O), mean ± SD	8.3 ± 3.3	7.1 ± 2.1*	7.8 ± 2.8	**<0.0001**
PIP (cmH_2O), mean ± SD	26.7 ± 8.1	17.2 ± 6.7*	21.7 ± 8.6*†	**<0.0001**
Plateau pressure (cmH_2O)[a], mean ± SD	23.0 ± 6.1	-	23.4 ± 6.4	0.7175
Patients with spontaneous ventilation (triggering ventilator), n (%)[b]	940 (51.1)	209 (99.1)*	109 (77.3)*†	**<0.0001**
**Immunocompromised patients (n=584)**				
Number of patients	462	63	59	
FiO_2, median (Q_1-Q_3)	0.6 (0.5-1.0)	0.6 (0.5-0.8)	0.6 (0.5-1.0)	0.5694
Set respiratory rate (bpm), mean ± SD	19.5 ± 7.1	11.0 ± 6.4*	18.8 ± 6.7†	**<0.0001**
Total respiratory rate (bpm), mean ± SD	22.0 ± 7.3	25.9 ± 5.0*	26.2 ± 7.6*	**<0.0001**
Tidal volume (mL/kg IBW), mean ± SD	7.5 ± 1.8	8.5 ± 2.3*	7.7 ± 2.8	**0.0289**
PEEP (cmH_2O), mean ± SD	8.6 ± 3.4	7.4 ± 1.9*	8.1 ± 2.6	**0.0323**
PIP (cmH_2O), mean ± SD	27.6 ± 8.8	14.9 ± 5.2*	22.8 ± 9.6*†	**<0.0001**
Plateau pressure (cmH_2O)[a], mean ± SD	23.0 ± 6.1	-	23.4 ± 6.4	0.6134
Patients with spontaneous ventilation (triggering ventilator), n (%)[b]	238 (52.31)	62 (100.0)	42 (71.2)	**<0.0001**

Abbreviations: IBW: ideal body weight; IMV: patients invasively ventilated from Day 1, independently of the type of support received after the eventual extubation; NIV: patients treated exclusively with non-invasive ventilation, from Day 1 to study exit, independently of outcome; NIV failure: patients initially treated with non-invasive ventilation and subsequently intubated during the study period; PEEP: positive end-expiratory pressure; PIP: peak inspiratory pressure; Q_1: first quartile; Q_3: third quartile; SD: standard deviation.

a. Plateau pressure were available for 571 immunocompetent patients (550 IMV and 21 NIV failure) and 171 immunocompromised patients (159 IMV and 12 NIV failure)

b. Information was available for 2191 immunocompetent patients (1839 IMV, 211 NIV and 141 NIV failure) and 576 immunocompromised patients (455 IMV, 62 NIV and 59 NIV failure)

Note: Bold p values shows a statistically significant difference between the two groups
