# Supplementary material for: Immunocompromised patients with acute respiratory distress syndrome: secondary analysis of the LUNG SAFE database
Source: Crit Care. 2018 Jun 12;22:157. doi: 10.1186/s13054-018-2079-9 (PMC5998562; doi:10.1186/s13054-018-2079-9)
Supplement: Supplementary file 7 — Figure S1. Kaplan-Meier curve for hospital survival in immunocompromised patients according to ARDS severity. Kaplan-Meier curve for hospital survival in immunocompromised patients according to ARDS severity. Mortality is defined as mortality at hospital discharge or at 90 days after onset of acute hypoxemic respiratory failure, whichever event occurred first. We assumed that patients discharged alive from the hospital before 90 days were alive on day 90. Severity of ARDS was evaluated at the day of onset according to the Berlin definition. Note: The number of patients reported in the bottom of figure is referred to as the end of the corresponding day. (PDF 402 kb) [file 13054_2018_2079_MOESM7_ESM.pdf]

Figure S1: Kaplan Meier curve for hospital survival of immunocompromised patients according to ARDS severity

Mortality is defined as mortality at hospital discharge or at 90 days after onset of acute hypoxemic respiratory failure, which ever event occurred first. We assumed that patients discharged alive from hospital before 90 days were alive on day 90. Severity of acute respiratory distress syndrome (ARDS) was evaluated at day of onset, according to the Berlin definition.

Note: the number of patients reported in the bottom of figure is referred to the end of corresponding day.

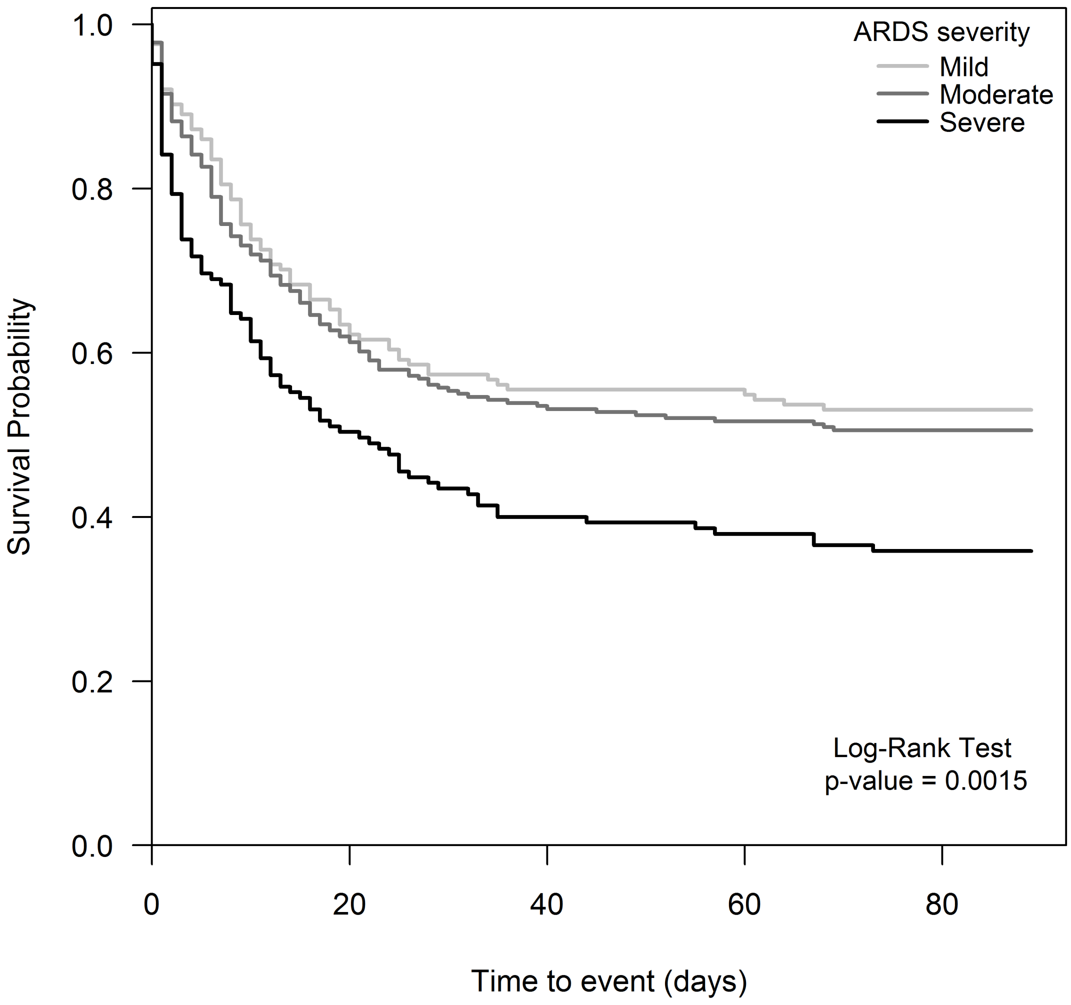

# at risk					
Mild	164	104	91	91	87
Moderate	271	168	145	140	137
Severe	145	73	58	55	52
