# Supplementary material for: Immunocompromised patients with acute respiratory distress syndrome: secondary analysis of the LUNG SAFE database
Source: Crit Care. 2018 Jun 12;22:157. doi: 10.1186/s13054-018-2079-9 (PMC5998562; doi:10.1186/s13054-018-2079-9)
Supplement: Supplementary file 8 — Figure S2. This figure shows a Kaplan-Meier curve for hospital survival of immunocompromised patients according to the ventilation subgroup. This figure shows a Kaplan-Meier curve for hospital survival of immunocompromised patients according to the ventilation subgroup. Mortality is defined as mortality at hospital discharge or at 90 days after onset of acute hypoxemic respiratory failure, whichever event occurred first. We assumed that patients discharged alive from the hospital before 90 days were alive on day 90. Type of ventilator support: IMV Patients invasively ventilated from day 1, independently of the type of support received after the eventual extubation; NIV Patients treated exclusively with noninvasive ventilation, from day 1 to study exit, independently of outcome; NIV failure Patients initially treated with noninvasive ventilation and subsequently intubated during the study period. Note: The number of patients reported in the bottom of the figure is referred to as the end of the corresponding day. (PDF 396 kb) [file 13054_2018_2079_MOESM8_ESM.pdf]

Figure S2. Kaplan Meier curve for hospital survival of immunocompromised patients according to the ventilation subgroup

Mortality is defined as mortality at hospital discharge or at 90 days after onset of acute hypoxemic respiratory failure, which ever event occurred first. We assumed that patients discharged alive from hospital before 90 days were alive on day 90.

Type of ventilator support: IMV: patients invasively ventilated from Day 1, independently of the type of support received after the eventual extubation; NIV: patients treated exclusively with non-invasive ventilation, from Day 1 to study exit, independently of outcome; NIV failure: patients initially treated with non-invasive ventilation and subsequently intubated during the study period.

Note: the number of patients reported in the bottom of figure is referred to the end of corresponding day.

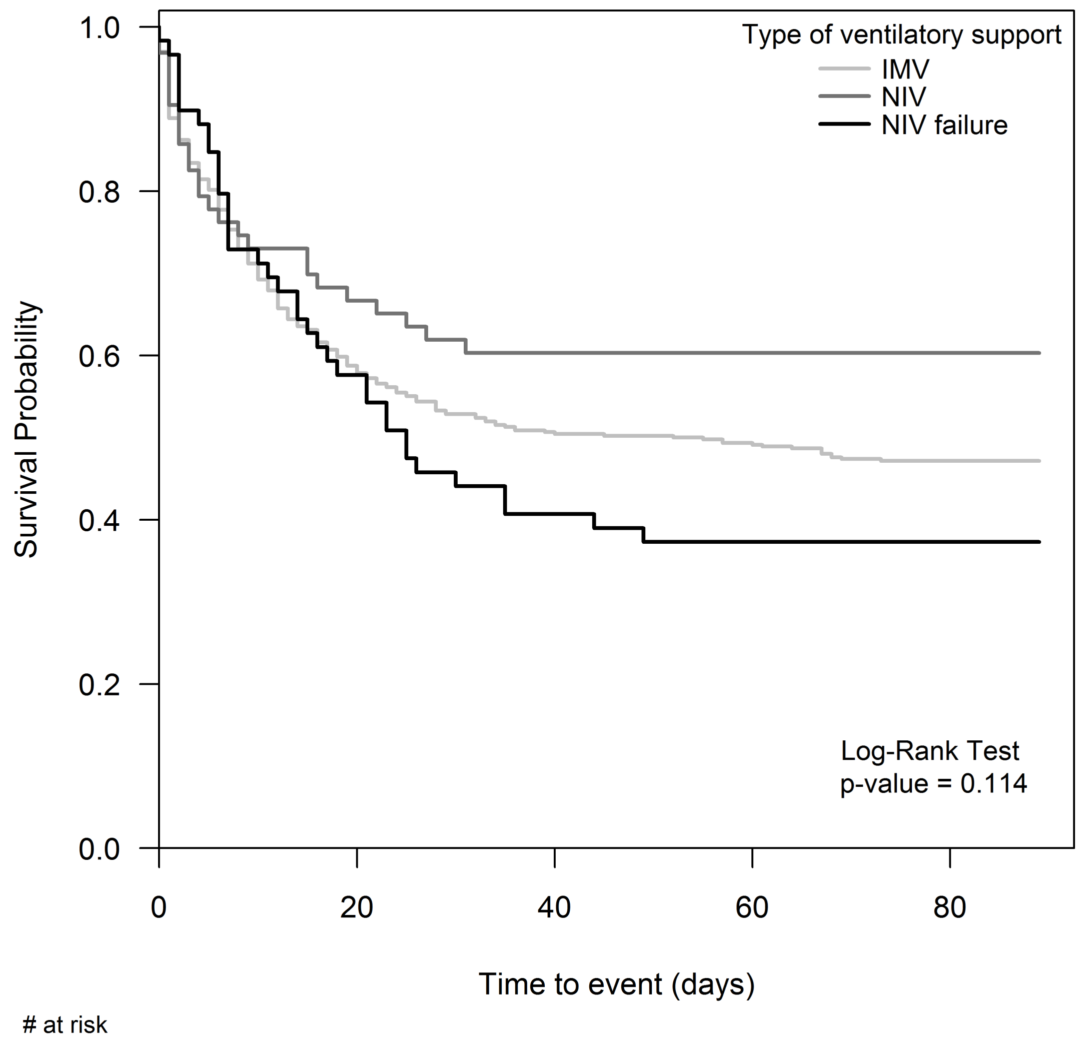

# at risk					
IMV	458	269	232	226	216
NIV	63	42	38	38	38
NIV failure	59	34	24	22	22
