# Supplementary material for: Immunocompromised patients with acute respiratory distress syndrome: secondary analysis of the LUNG SAFE database
Source: Crit Care. 2018 Jun 12;22:157. doi: 10.1186/s13054-018-2079-9 (PMC5998562; doi:10.1186/s13054-018-2079-9)
Supplement: Supplementary file 9 — Table S5. Factors associated with hospital mortality in immunocompromised patients. Multivariate logistic regression model describing the factors associated with hospital mortality in immunocompromised patients. (PDF 49 kb) [file 13054_2018_2079_MOESM9_ESM.pdf]

Table S5: Multivariable logistic regression model: factors associated with hospital mortality in immunocompromised (Study) patients (n=463).

Effect	OR	95% CI		P value
Non-pulmonary SOFA score[a]	1.079	1.026	1.134	0.0032
P/F ratio (mmHg)	0.995	0.992	0.998	0.0022
Δ P/F ratio (%)[b]	0.996	0.993	0.999	0.0058
BMI (kg/m^2)	0.944	0.910	0.980	0.0023
PIP (cm H_2O)	1.028	1.007	1.051	0.0097

Abbreviations: BMI: body mass index; CI: confidence interval; OR: odds ratio; PIP: peak inspiratory pressure; SOFA: sequential organ failure assessment

a. Non pulmonary SOFA score adjusted for missing values

b. Delta (Δ) was evaluated as difference between the value measured at the second day and those measured at the ARDS onset day. Δ (%) was evaluated as rate between Δ and value measured at the ARDS onset day

Note: Mortality is defined as mortality at hospital discharge or at ninetieth day in hospital, after onset of acute hypoxemic respiratory failure, which ever event occurred first.
