# Supplementary material for: Immunocompromised patients with acute respiratory distress syndrome: secondary analysis of the LUNG SAFE database
Source: Crit Care. 2018 Jun 12;22:157. doi: 10.1186/s13054-018-2079-9 (PMC5998562; doi:10.1186/s13054-018-2079-9)
Supplement: Supplementary file 10 — Table S9. The most important factors leading to death in the ICU in immunocompetent and immunocompromised patients. (PDF 44 kb) [file 13054_2018_2079_MOESM10_ESM.pdf]

Table S9: Most important factors leading to death in ICU, in immunocompetent (Control) and immunocompromised (Study) patients

	**Control (n=698)**	**Study (n=266)**	**p value**
Respiratory failure, n (%)	264 (37.8)	137 (51.5)	**0.0001**
Cardiovascular failure, n (%)	271 (38.8)	87 (32.7)	0.0788
Neurologic failure, n (%)	85 (12.2)	20 (7.5)	**0.0380**
Hepatic failure, n (%)	42 (6.0)	9 (3.4)	0.1025
Renal failure, n (%)	21 (3.0)	7 (2.6)	0.7554
Coagulation failure, n (%)	10 (1.4)	2 (0.8)	0.5277
Unknown, n (%)	5 (0.7)	4 (1.5)	0.2704
