# Supplementary material for: Immunocompromised patients with acute respiratory distress syndrome: secondary analysis of the LUNG SAFE database
Source: Crit Care. 2018 Jun 12;22:157. doi: 10.1186/s13054-018-2079-9 (PMC5998562; doi:10.1186/s13054-018-2079-9)
Supplement: Supplementary file 11 — Table S7. Patient characteristics and clinical endpoints of immunocompetent patients, according to the type of ventilatory support. (PDF 88 kb) [file 13054_2018_2079_MOESM11_ESM.pdf]

**Table S7: Patient characteristics and clinical endpoints of immunocompetent (Control) patients according to the type of ventilatory support**

| | IMV (n=1,874) | NIV (n=212) | NIV failure (n=143) | p value |
|---|---|---|---|---|
| **Patient characteristics** | | | | |
| ***Sex, age and BMI*** | | | | |
| Women, n (%) | 685 (36.6) | 92 (43.4) | 60 (42.0) | 0.0793 |
| Age (years), mean ± SD | 60.8 ± 17.2 | 67.7 ± 16.3* | 62.1 ± 15.2† | **<0.0001** |
| BMI (kg/m$^2$), mean ± SD | 28.0 ± 9.3 | 28.3 ± 8.3 | 28.8 ± 10.4 | 0.9083 |
| ***Co-morbidities, n (%)*** | | | | |
| COPD | 398 (21.2) | 77 (36.3)* | 47 (32.9)* | **<.0001** |
| Diabetes mellitus | 436 (23.3) | 59 (27.8) | 28 (19.6) | 0.1744 |
| Hearth failure (NYHA classes III-IV) | 202 (10.8) | 37 (17.5)* | 21 (14.7) | **0.0083** |
| Chronic renal failure | 172 (9.2) | 36 (17.0)* | 14 (9.8) | **0.0016** |
| Chronic liver failure (Child-Pugh Class C) | 81 (4.3) | 6 (2.8) | 4 (2.8) | 0.4216 |
| Home ventilation | 33 (1.8) | 11 (5.2)* | 8 (5.6)* | **0.0004** |
| ***ARDS risk factors, n (%)*** | | | | |
| Pneumonia | 1035 (55.2) | 129 (60.8) | 107 (74.8)*† | **<0.0001** |
| Pulmonary contusion | 75 (4.0) | 4 (1.9) | 7 (4.9) | 0.2541 |
| Pulmonary vasculitis | 5 (0.3) | 1 (0.5) | 1 (0.7) | 0.3094 |
| Major trauma | 102 (5.4) | 3 (1.4)* | 6 (4.2) | **0.0346** |
| Aspiration of gastric contents | 322 (17.2) | 18 (8.5)* | 8 (5.6)* | **<0.0001** |
| Pancreatitis | 45 (2.4) | 7 (3.3) | 2 (1.4) | 0.5139 |
| Non-cardiogenic shock | 146 (7.8) | 2 (0.9)* | 6 (4.2) | **0.0004** |
| Drug-overdose | 44 (2.3) | 1 (0.5) | 1 (0.7) | 0.0987 |
| Severe burns | 8 (0.4) | 0 (0.0) | 0 (0.0) | 1.0000 |
| Inhalational injury | 55 (2.9) | 1 (0.5) | 2 (1.4) | 0.0661 |
| Drowning | 1 (0.1) | 0 (0.0) | 0 (0.0) | 1.0000 |
| Non-pulmonary sepsis | 327 (17.4) | 21 (9.9)* | 13 (9.1)* | **0.0011** |
| Blood transfusions | 74 (3.9) | 4 (1.9) | 4 (2.8) | 0.2698 |
| Other risk factors | 58 (3.1) | 2 (0.9) | 1 (0.7) | 0.0580 |
| None | 146 (7.8) | 39 (18.4)* | 9 (6.3)† | **<0.0001** |
| ***Illness severity at ARDS onset*** | | | | |
| Non-pulmonary SOFA score[a], mean ± SD | 6.8 ± 4.0 | 2.8 ± 2.9* | 4.5 ± 4.0*† | **<0.0001** |
| $PaO_2/FiO_2$ ratio (mm Hg), mean ± SD | 162.1 ± 67.6 | 166.8 ± 65.1 | 142.8 ± 59.9*† | **0.0018** |
| Mild ARDS[b], n (%) | 570 (30.4) | 70 (33.0) | 26 (18.2)*† | **0.0050** |
| Moderate ARDS[b], n (%) | 889 (47.4) | 102 (48.1) | 76 (53.1) | 0.4189 |
| Severe ARDS[b], n (%) | 415 (22.2) | 40 (18.9) | 41 (28.7) | 0.0897 |
| **Clinical endpoints** | | | | |
| ***Duration of mechanical ventilation (days), median ($Q_1$-$Q_3$)*** | 8.0 (4.0-16.0) | - | 9.0 (4.0-13.0) | 0.9345 |
| ***Progression/Regression of ARDS[c], n (%)*** | | | | |
| No change | 697 (41.5) | 60 (34.9) | 67 (52.8)*† | **0.0078** |
| Progression | 187 (11.1) | 15 (8.7) | 12 (9.4) | 0.5465 |
| Regression | 378 (22.5) | 24 (14.0)* | 20 (15.7) | **0.0094** |
| Resolution | 417 (24.8) | 73 (42.4)* | 28 (22.0)† | **<.0001** |
| ***Limitation of life sustaining measures, n (%)*** | | | | |
| Decision to withhold life sustaining measures | 338 (18.0) | 50 (23.6) | 27 (18.9) | 0.1439 |
| Decision to withdraw life sustaining measures | 303 (16.2) | 32 (15.1) | 21 (14.7) | 0.8386 |
| Decision to withhold or withdraw life sustaining measures | 417 (22.3) | 57 (26.9) | 33 (23.1) | 0.3107 |
| Before IMV or NIV start | 3 (0.7) | 2 (3.5) | 0 (0.0) | 0.1344 |
| ***ICU mortality[d], n (%)*** | 609 (32.5) | 40 (18.9)* | 49 (34.4)† | **0.0002** |
| ***Hospital mortality[e], n (%)*** | | | | |
| All patients | 693 (37.1) | 60 (28.3)* | 51 (35.9) | **0.0416** |
| Patients with limitations of life sustaining measures[d] | 354 (84.9) | 38 (66.7)* | 27 (81.8) | **0.0030** |

Abbreviations: ARDS: acute respiratory distress syndrome; COPD: chronic obstructive pulmonary disease; IMV: patients invasively ventilated from Day 1, independently of the type of support received after the eventual extubation; NIV: patients treated exclusively with non-invasive ventilation, from Day 1 to study exit, independently of outcome; NIV failure: patients initially treated with non-invasive ventilation and subsequently intubated during the study period; NYHA: New York Heart Association; $Q_1$: first quartile; $Q_3$: third quartile; SD: standard deviation; SOFA: sequential organ failure assessment.

a. Non pulmonary SOFA score adjusted for missing values

b. Severity of ARDS was evaluated according to the Berlin definition

c. Change in ARDS severity (according Berlin definition) was not evaluable for 251 immunocompetent patients (195 IMV, 40 NIV and 16 NIV failure).

d. Mortality is defined as mortality at ICU discharge or at ninetieth day in ICU, after onset of acute hypoxemic respiratory failure, which ever event occurred first

e. Mortality is defined as mortality at hospital discharge or at ninetieth day in hospital, after onset of acute hypoxemic respiratory failure, which ever event occurred first.

Note: Bold p values shows a statistically significant difference among the three groups

* Statistically significant different from IMV group; † Statistically significant different from NIV group
