# Supplementary material for: Immunocompromised patients with acute respiratory distress syndrome: secondary analysis of the LUNG SAFE database
Source: Crit Care. 2018 Jun 12;22:157. doi: 10.1186/s13054-018-2079-9 (PMC5998562; doi:10.1186/s13054-018-2079-9)
Supplement: Supplementary file 12 — Table S8. Patients’ characteristics and clinical endpoints of immunocompromised (study) patients, according to the cause of immunosuppression (known, unknown). (PDF 79 kb) [file 13054_2018_2079_MOESM12_ESM.pdf]

Table S8: Patient characteristics and clinical endpoints of immunocompromised (Study) patients according to the cause of immunosuppression (known, unknown)

	Cause known (n=357)	Cause unknown (n=227)	p value
**Patient characteristics**			
Sex, age and BMI			
Women, n (%)	137 (38.4)	110 (48.5)	**0.0162**
Age (years), mean ± SD	63.1 ± 14.8	55.7 ± 15.6	**<.0001**
BMI (kg/m^2), mean ± SD	25.6 ± 5.7	25.2 ± 6.0	0.1220
Co-morbidities, n (%)			
COPD	54 (15.1)	31 (13.7)	0.6235
Diabetes mellitus	49 (13.7)	41 (18.1)	0.1571
Hearth failure (NYHA classes III-IV)	16 (4.5)	14 (6.2)	0.3684
Chronic renal failure	33 (9.2)	31 (13.7)	0.0961
Chronic liver failure (Child-Pugh Class C)	9 (2.5)	12 (5.3)	0.0802
Home ventilation	3 (0.8)	4 (1.8)	0.4394
ARDS risk factors, n (%)			
Pneumonia	236 (66.1)	176 (77.5)	**0.0031**
Pulmonary contusion	1 (0.3)	0 (0.0)	1.0000
Pulmonary vasculitis	0 (0.00)	7 (3.1)	**0.0013**
Major trauma	1 (0.3)	0 (0.0)	1.0000
Aspiration of gastric contents	45 (12.6)	9 (4.0)	**0.0004**
Pancreatitis	5 (1.4)	0 (0.0)	0.1622
Non-cardiogenic shock	40 (11.2)	20 (8.8)	0.3530
Drug-overdose	2 (0.6)	3 (1.3)	0.3819
Severe burns	0 (0.00)	0 (0.00)	-
Inhalational injury	9 (2.5)	3 (1.3)	0.3843
Drowning	1 (0.3)	0 (0.0)	1.0000
Non-pulmonary sepsis	60 (16.8)	34 (15.0)	0.5577
Blood transfusions	22 (6.2)	7 (3.1)	0.0950
Other risk factors	12 (3.4)	1 (0.4)	**0.0197**
None	26 (7.3)	14 (6.2)	0.6029
Illness severity at ARDS onset			
Non-pulmonary SOFA score[a], mean ± SD	6.8 ± 3.9	6.0 ± 4.0	**0.0200**
PaO_2/FiO_2 ratio (mm Hg), mean ± SD	162.8 ± 68.7	148.3 ± 65.9	**0.0146**
Mild ARDS[b], n (%)	115 (32.2)	52 (22.9)	**0.0153**
Moderate ARDS[b], n (%)	164 (45.9)	107 (47.1)	0.7772
Severe ARDS[b], n (%)	78 (21.9)	68 (30.0)	**0.0274**
**Clinical endpoints**			
IMV during ICU stay, n (%)	283 (79.3)	179 (78.9)	0.9038
NIV success during ICU stay, n (%)	37 (10.4)	26 (11.4)	0.6791
NIV failure during ICU stay, n (%)	37 (10.4)	22 (9.7)	0.7926
Duration of mechanical ventilation (days), median (Q_1-Q_3)	7.0 (4.0-13.0)	9.0 (4.0-17.0)	0.0531
Progression/Regression of ARDS[c], n (%)			0.3904
No change	123 (34.5)	78 (34.4)	
Progression	35 (9.8)	20 (8.8)	
Regression	67 (18.8)	56 (24.7)	
Resolution	86 (24.1)	43 (18.9)	

Limitation of life sustaining measures, n (%)			
Decision to withhold life sustaining measures	108 (30.3)	50 (22.0)	**0.0292**
Decision to withdraw life sustaining measures	92 (25.8)	37 (16.3)	**0.0072**
Decision to withhold or withdraw life sustaining measures	133 (37.3)	62 (27.3)	**0.0130**
Before IMV or NIV start	1 (0.8)	1 (1.6)	0.5359
ICU mortality[d], n (%)	180 (50.4)	86 (37.9)	**0.0030**
Hospital mortality[e], n (%)			
All patients	210 (59.3)	94 (41.6)	**<.0001**
Patients with limitations of life sustaining measures[d]	119 (89.5)	54 (87.1)	0.6252

Abbreviations: ARDS: acute respiratory distress syndrome; COPD: chronic obstructive pulmonary disease; IMV: patients invasively ventilated from Day 1, independently of the type of support received after the eventual extubation; NIV: patients treated exclusively with non-invasive ventilation, from Day 1 to study exit, independently of outcome; NIV failure: patients initially treated with non-invasive ventilation and subsequently intubated during the study period; NYHA: New York Heart Association; Q_1: first quartile; Q_3: third quartile; SD: standard deviation; SOFA: sequential organ failure assessment.

a. Non pulmonary SOFA score adjusted for missing values

b. Severity of ARDS was evaluated according to the Berlin definition

c. Change in ARDS severity (according Berlin definition) was not evaluable for 76 immunocompromised patients (43 with cause known, 30 with cause unknown).

d. Mortality is defined as mortality at ICU discharge or at ninetieth day in ICU, after onset of acute hypoxemic respiratory failure, which ever event occurred first

e. Mortality is defined as mortality at hospital discharge or at ninetieth day in hospital, after onset of acute hypoxemic respiratory failure, which ever event occurred first.

Note: Bold p values shows a statistically significant difference among the three groups
